# Amyloid beta plaque pathology induces chemosensory deficits and increases apnoeas in the 5XFAD mouse model of Alzheimer's disease

**DOI:** 10.1113/JP290936

**Published:** 2026-09-05

**Authors:** Ryan J. Sprenger, Andrea C. Ewald, Phinea Z. Romero, Kaitlyn M. Marino, Maia G. Gumnit, Erin C. McCann, Abigail B. Radcliff, Tyler K. Ulland, Jyoti J. Watters, Tracy L. Baker

**Affiliations:** ^1^ Department of Comparative Biosciences University of Wisconsin‐Madison Madison Wisconsin USA; ^2^ Department of Pathology and Laboratory Medicine University of Wisconsin Madison Wisconsin USA; ^3^ Neuroscience Training Program University of Wisconsin Madison Wisconsin USA; ^4^ Wisconsin Alzheimer's Disease Research Center University of Wisconsin Madison Wisconsin USA

**Keywords:** Alzheimer's disease, amyloid beta, chemosensory, respiration

## Abstract

**Abstract:**

Sleep disordered breathing (SDB) commonly co‐presents with other ventilatory impairments in patients with Alzheimer's disease (AD). Typically reported as either obstructive (OSA) or complex sleep apnoea in AD patients, SDB results in repeated bouts of hypoxia and hypercapnia from insufficient ventilation. It is thought not only that these repeated hypoxic events result in an exacerbation of AD but also that SDB and other ventilatory impairments may be a precursor to neurodegenerative diseases. Although the cause of SDB in AD patients is unclear, chemosensory drive malfunction, a response critical to maintaining homeostatic oxygen and carbon dioxide balance, may play a role. We examined basal ventilation, SDB and chemosensory function in 5XFAD mice, a beta amyloidosis model of AD which displays progressive amyloid beta (Aβ) plaque deposition by 8 months of age. We found that both male and female 5XFAD mice had nearly twice as many spontaneous apnoeas per hour compared to WT mice. Basal ventilation (*V̇*_E_) and air convection requirement (ACR) while awake were unchanged, but both hypoxic and hypercapnic ventilatory responses were impaired in 5XFAD mice. While WT mice responded to acute hypoxia with the expected increase in *V̇*_E_ of ∼150%, 5XFAD mice showed a blunted increase of ∼70% with a similarly blunted hypercapnic response (∼100% increase in 5XFAD mice/∼200% increase in WT mice). Further, we found substantial Aβ deposition in the ventral lateral medulla, an important site for rhythm generation and chemosensing. Taken together, these data suggest a correlation between SDB, Aβ deposition and chemosensory deficits in 5XFAD mice.

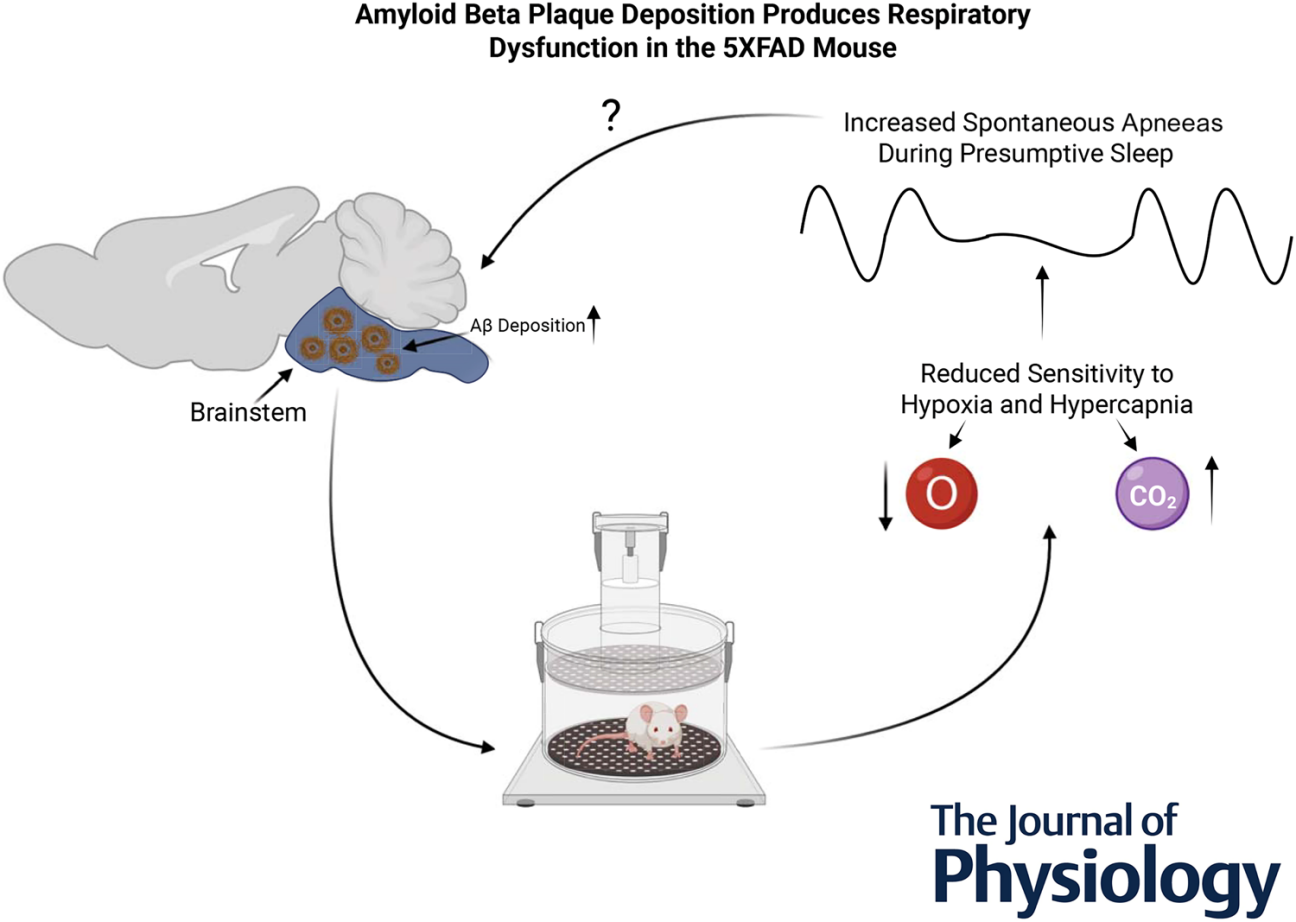

**Key points:**

- Respiratory dysfunction is common among Alzheimer's disease (AD) patients, but the nature of this dysfunction is not well understood
- Sleep disordered breathing (SDB) is prevalent in AD patients and is thought to contribute to disease progression
- 5XFAD mice, a model of beta amyloidosis, display similar respiratory deficits to humans with AD, including SDB and chemosensory changes
- We show that amyloid beta plaque deposition in the brainstem of 5XFAD mice localizes to regions of respiratory control
- Our data suggest that amyloid beta plaque deposition in brainstem respiratory control regions contributes to chemosensory dysfunction in 5XFAD mice, a pathology that could worsen SDB occurrence and potentially Aβ‐dependent neuropathologies such as AD

## Graphical abstract

**Abstract figure legend** Amyloid beta deposition in 5XFAD mice produces respiratory dysfunction. Amyloid beta (Aβ) plaque deposition in respiratory related regions of the brainstem was observed in 5XFAD mice, which also displayed significant reductions in their hypercapnic and hypoxic ventilatory responses. Concurrently, 5XFAD mice exhibited a significant rise in spontaneous apnoeas during presumptive sleep. The interaction between apnoeas and Aβ plaque deposition is unclear but may be involved in exacerbation of neuropathology and worsening of respiratory function.

## Introduction

Alzheimer's disease (AD) is the most common cause of dementia world‐wide. Increasing age is the largest risk factor for developing AD. While the majority of AD cases are late onset and sporadic with causes mostly unknown, familial early onset disease accounts for approximately 5% of all cases (Daulatzai, 2013).

Besides ageing, other risk factors also appear to contribute to AD onset and progression (Daulatzai, 2013; Kazim et al., 2021; Sun et al., 2006) including repeated intermittent hypoxia exposure (Daulatzai, 2013; Kazim et al., 2021; Leng et al., 2017; Macheda et al., 2019; Sun et al., 2006; Wang et al., 2024; Yaffe et al., 2012). Approximately 70% of institutionalized AD patients show respiratory changes (Daulatzai, 2013; Gaeta et al., 2020), including 45% with sleep disturbances including apnoeas, insufficient ventilation during sleep and shortness of breath (Gaeta et al., 2020; Ogbu et al., 2024). Obstructive sleep apnoea (OSA) is the most commonly reported form of sleep disordered breathing (SDB), and it results from both loss of airway patency and insufficient compensatory respiratory drive, whereas central sleep apnoea (CSA) arises from dysfunction in respiratory drive most often due to hypersensitivity in chemosensory feedback and central respiratory function (Deak & Kirsch, 2014; Dempsey, 2019; Gaeta et al., 2020). Often both OSA and CSA co‐exist in patients, in a condition referred to as complex sleep apnoea (Daulatzai, 2013; Deak & Kirsch, 2014). Although the mechanisms producing the intermittent hypoxia associated with OSA and CSA in AD are not yet fully understood, growing evidence implicates hypoxia in both AD pathology initiation and progression (Kazim et al., 2021; Li et al., 2009; Sun et al., 2006; Wrzesień et al., 2024; Zhang et al., 2013). Intermittent hypoxia exacerbates AD symptom progression in the 3xTg AD mouse model of amyloid beta (Aβ) plaque deposition (Shiota et al., 2013).

There is a growing appreciation for chemosensory function and disease pathogenesis in multiple diseases (Babiloni et al., 2014; Burtscher et al., 2024; Cabot et al., 2026; Correia & Moreira, 2022; Erhardt et al., 2025; Mulkey et al., 2025; Pokusa et al., 2020; Rey et al., 2023), and these data suggest that chemosensory dysfunction may contribute to AD progression by increasing the occurrence of SDB which worsens AD pathology (Dempsey, 2019; Gaig & Iranzo, 2012; Humphrey et al., 2023; Iseki et al., 1989; Orr et al., 2017; Simic et al., 2009). In the interest of better understanding the interaction of chemosensory dysfunction and AD pathogenesis, we tested the hypothesis that chemosensory dysfunction accompanies apnoeas early in AD‐like pathology. We utilized the 5XFAD mouse model of early/beta amyloid centric AD pathology to examine the co‐occurrence of SDB and chemosensory dysfunction in middle‐aged (8 month) adult mice. We also examined the deposition of Aβ plaques in central respiratory control regions to determine whether plaque load correlates with respiratory impairments. We found that 5XFAD mice had increased occurrences of apnoeic events during presumptive sleep, and deficiencies in their hypercapnic and hypoxic ventilatory responses. Amyloid plaque load correlated with decreased hypercapnic but not hypoxic ventilatory responses. Together these results suggest that medullary chemosensory function is altered by Aβ plaque deposition early in pathogenesis and may contribute to the occurrence of SDB in 5XFAD mice by producing deficits in hypercapnic feedback corrections.

## Methods

### Animal housing, care and ethical approval

The following study adheres to the *Journal of Physiology* policies for animal experiments. Wild‐type (WT; C57BL/6J) and 5XFAD (Tg6799) mice were obtained from the Jackson Laboratory (Bar Harbor, ME, USA) and housed in an AAALAC‐accredited vivarium. Food and water were available *ad libitum*. Animals were group housed (two per cage) in standard mouse cages with sani‐chip bedding and tek‐fresh/enviro‐dri nesting material (Teklad) with PVC tubes for enrichment. Room temperatures were maintained at ∼24°C with relative humidity maintained at 20–40%. All procedures were conducted under protocols approved by University of Wisconsin Animal Care and Use Committee (V005173).

### Number of animals

For all ventilatory measurements *n* = 7 (WT male), 8 (WT female), 11 (5XFAD male) and 12 (5XFAD female). For all sleep measurements *n* = 10 for all groups. For all medullary staining measurements *n* = 8 (5XAFD males) and 7 (5XFAD females). For all pontine staining measurements *n* = 8 (5XFAD males) and 6 (5XFAD females). We also added three male and four female WT animals to all staining protocols.

### Respiratory measurements

Ventilatory patterns and metabolic variables were continuously measured using flow through whole‐body plethysmography as described previously (Data Sciences International, Minneapolis, MN, USA) (Drorbaugh & Fenn, 1955; Jacky, 1978; Sprenger & Milsom, 2021a, 2021b; Sprenger et al., 2019). Mice were placed in plethysmograph chambers between 12 and 4 pm each day.

### Experimental protocol

Throughout the experiments O_2_ consumption (${\dot{V}}_{O_{2}}$), CO_2_ production, breathing frequency (*f*), and tidal volume (*V*_T_) were measured continuously. Body temperature (*T*_B_) was periodically measured (∼5 min intervals) using implantable radio frequency identification chips (ITPP‐300) as we previously reported (Sprenger & Milsom, 2021b). Inhaled 2% isoflurane was given to animals prior to syringe implantation of the ITPP‐300 RFID chips, a procedure taking less than 2 min in total. All animals were given 30 min in room air to acclimate to the chambers prior to challenges. We measured the hypoxic ventilatory response (HVR; 5% O_2_/0% CO_2_ for 5 min), the hypoxia + 5% CO_2_ ventilatory response (H+5; 5% O_2_/5% CO_2_ for 5 min), the hypercapnic ventilatory response (HCVR; 21% O_2_/0% CO_2_; 21% O_2_/1% CO_2_; 21% O_2_/3% CO_2_; 21% O_2_/5% CO_2_ at 20 min per step) and the maximal hypercapnia/hypoxia response (8% O_2_/5% CO_2_ for 10 min). The HCVR curve was performed sequentially in all animals to reduce time in the chamber. At the end of the study, animals were killed via isoflurane overdose followed by decapitation.

### Immunostaining and quantification

Brainstems were postfixed in 4% paraformaldehyde before being frozen. For respiratory region analysis, serial coronal brainstem sections (20 µm) were cut using a cryostat. Brainstems were stained with anti‐tyrosine hydroxylase antibodies (Abcam, ab76442, Cambridge, UK) to identify the locus coeruleus (LC). The ventrolateral medulla (VLM) location was determined by proximity to hypoglossal nucleus (VII) and the nucleus tractus solitarius (NTS). Aβ plaques were stained using methoxy‐X04 (Tocris Bioscience, 4920, Minneapolis, MN, USA). Images were taken on a THUNDER Imager (Leica Microsystems Inc.). Z‐stack images were compressed to one final image. Plaque quantity and size were analysed using FIJI (ImageJ). Plaques were counted following pixel thresholding and areas larger than 65 pixels were included. Plaque numbers from three slices per animal were counted unilaterally (both left and right) and then added and averaged in each respiratory region.

### Data analysis

**Figure 1:**
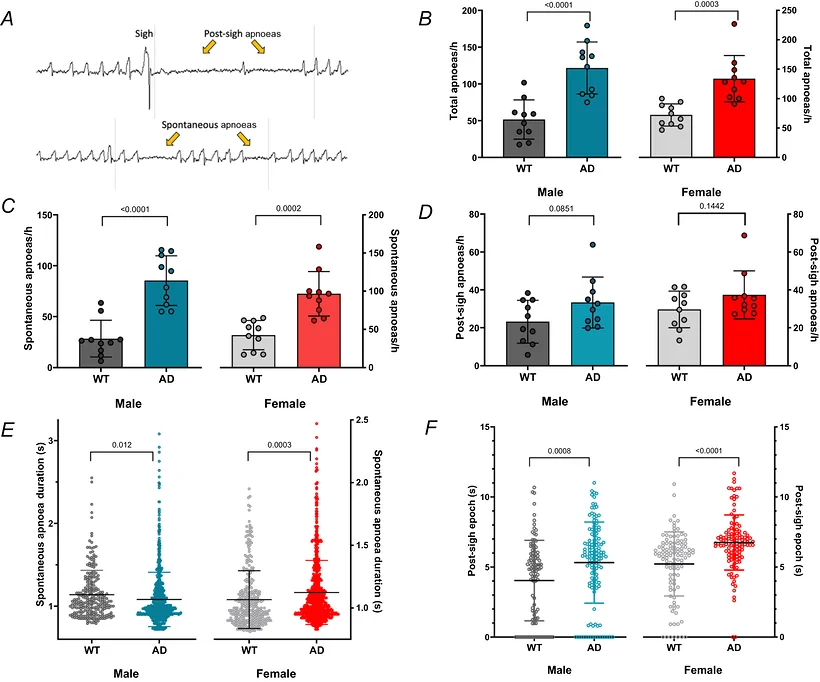
5XFAD mice have increased spontaneous apnoeas and apnoea durations *A*, representative breathing traces showing a sigh, post‐sigh and spontaneous apnoeas. *B*–*F*, the number of total apnoeas (*B*), and spontaneous apnoeas/h (*C*), post‐sigh apnoeas/h (*D*), spontaneous apnoeas (*E*), and post‐sigh epochs (*F*) in wildtype (WT) and 5XFAD (AD) mice. Data are presented as mean ± SD. Significant *P*‐values are noted in the figure (*P* < 0.05). Welch's *t* test; *n* = 10 for all groups.

All hypoxic and hypercapnic plethysmography data were collected using a Powerlab acquisition system (AD Instruments) and analysed using Labchart software (AD Instruments). Plethysmography traces used for sleep analysis (Fig. 1*A*) were acquired using DSI acquisition (Ponemah) and analysis software (Neuroscore). Presumptive sleep and wake periods in plethysmography traces were analysed as previously described (Fields et al., 2019). Apnoeas were defined as two or more skipped breaths. For post‐sigh apnoea analysis, we examined for apnoeas within a 10 s epoch following the sigh. During the wake periods, traces were analysed during the last 5 min of each exposure. For the HVR analysis, the maximum *f* and *V*_T_ were taken during the 5 min exposure period.

### Statistical methods

All statistical analyses were performed using GraphPad PRISM version 9.1.0.

For sleep data only, we used a ROUT test from Graphpad Prism allowing for multiple outliers to be identified. We used Q = 0.1 to identify definitive outliers. Approximately 2% of the apnoea durations were identified as outliers using this method, but we chose not to exclude those datapoints given that apnoeas are easily and definitively identified from the plethysmography data. No other outliers were identified. Welch's *t* tests were used to examine differences between wild type (WT) and 5XFAD mice given only two groups for each test.

For all ventilatory and metabolic responses to hypoxia and hypercapnia data, multiple un‐paired Student's *t* tests were used to examine differences between WT and 5XFAD mice using the Holm–Šídák correction for multiple comparisons given multiple exposures per test. For hypoxia + 5% CO_2_ responses only a two‐wayANOVA followed by the Holm–Šídák *post hoc* test was used.

For amyloid deposition and ventilatory response correlations, a simple linear regression with Pearson correlation coefficients was used to determine if the slope was significantly different from 0 and if there was a significant correlation.

Statistical significance was set at *P* < 0.05. All data are presented as the mean ± SD.

## Results

### 5XFAD mice have increased apnoeas during sleep

Apnoeas were measured by plethysmography and occurred either spontaneously during regular rhythmic breathing or following a sigh (Fig. 1*A*). Both male and female 5XFAD mice exhibited significantly more total apnoeas during presumptive sleep than WT animals (Fig. 1*B*) with 5XFAD males (121.6 ± 35.4 apnoeas/h; Fig. 1*D*, *P* = 0.0001) and females (133.8 ± 39.4 apnoeas/h; Fig. 1*B*, *P* = 0.0003) displaying more than double that of their WT counterparts (males: 51.6 ± 26.6; females: 72.1 ± 18.9).

Spontaneous apnoea frequency in 5XFAD males was 85.3 ± 24.4 apnoeas/h, nearly triple that observed in WT males (28.4 ± 18 apnoeas/h; Fig. 1*C*, *P* = 0.0001). Similarly, 5XFAD females exhibited 96.5 ± 29.2 apnoeas/h, more than double the number in WT females (42.5 ± 19 apnoeas/h; Fig. 1*C*, *P* = 0.0001). Interestingly, 5XFAD male mice had significantly shorter spontaneous apnoea durations than WT males (WT: 1.14 ± 0.29 s, 5XFAD: 1.08± 0.33 s; Fig. 1*E*, *P* = 0.012) whereas 5XFAD females had significantly longer spontaneous apnoea durations than WT females (5XFAD: 1.12 ± 0.26 s, WT: 1.07 ± 0.23 s; Fig. 1*E*, *P* = 0.0003).

The number of sighs did not differ between 5XFAD and WT animals in either sex (males: 5XFAD 14.7 ± 4.5 *vs*. WT 12.9 ± 3.8 sighs/h; females: 5XFAD 14.7 ± 2.7 *vs*. WT 13.9 ± 2.0 sighs/h) (Table A1). While the number of post‐sigh apnoeas did not reach statistical significance, there was a slight trend in 5XFAD mice towards an increase in the number of post‐sigh apnoeas in both sexes (males: 5XFAD 33.3 ± 13.4 *vs*. WT 23.2 ± 11.3 post‐sigh apnoeas/h, *P* = 0.085, Fig. 1*D*; females: 5XFAD 37.3 ± 12.7 *vs*. WT 29.6 ± 9.7 post‐sigh apnoeas/h, *P* = 0.144, Fig. 1*D*). Both male and female 5XFAD mice had significantly longer post‐sigh epoch durations compared to WT animals [*P* = 0.0011 (male), *P* = 0.0001 (female), Fig. 1*F*].

### 5XFAD mice display chemosensory deficits

The 5XFAD genotype had no effect on resting/awake ${\dot{V}}_{O_{2}}$, ${\dot{V}}_{E}$, air convection requirement (ACR) or *T*_B_ (Table A1) when all resting conditions were combined. ${\dot{V}}_{O_{2}}$ ranged between 0.045 and 0.06 ml/min/g in males and between 0.06 and 0.08 ml/min/g in females; ${\dot{V}}_{E}$ ranged between 1 and 1.5 ml/min/g in males and females (Table A1). Despite the small variation in both ${\dot{V}}_{E}$ and ${\dot{V}}_{O_{2}}$, ACR was consistently ∼16–18 among all animals at rest. Of note, resting ${\dot{V}}_{O_{2}}$ and ${\dot{V}}_{CO_{2}}$ were significantly elevated in 5XFAD males compared to WT males if data were split into their respective responses (hypoxia or hypercapnia), though this effect was not apparent if the animals were grouped at rest (Table A1).

**Figure 2:**
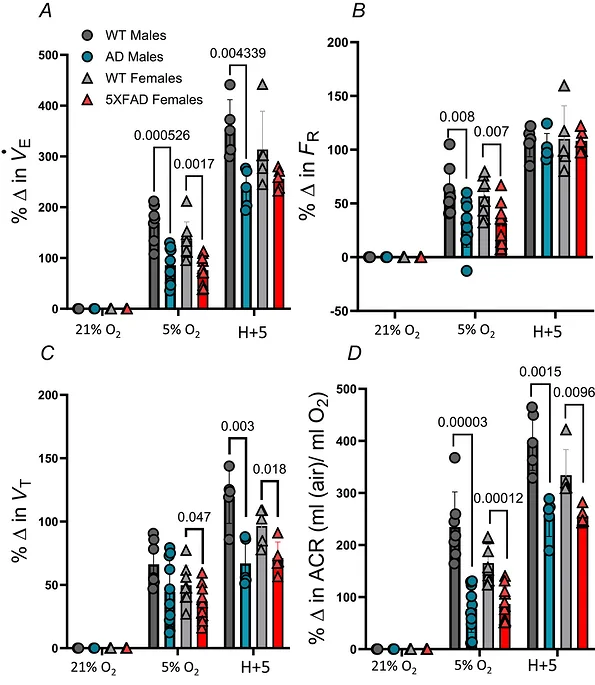
5XFAD mice have impaired ventilatory responses to hypoxia Changes in: *A*, ventilation (${\dot{V}}_{E}$); *B*, breathing frequency (*F*_R_); *C*, tidal volume (*V*_T_); and *D*, the air convection requirement (ACR) to acute 5% hypoxia were assessed in wild type (WT) and 5XFAD (AD) male and female mice. H+5 denotes hypoxic + 5% CO_2_ (5% CO_2_/8% O_2_) used to elicit a maximal ventilatory response. Error bars represent SD, and significant *P*‐values are noted in the figure (*P* < 0.05). Multiple Student's *t* tests with Holm–Šídák *post hoc* test; *n* = 7 (WT male), 8 (WT female), 11 (5XFAD male), and 12 (5XFAD female).

**Figure A1:**
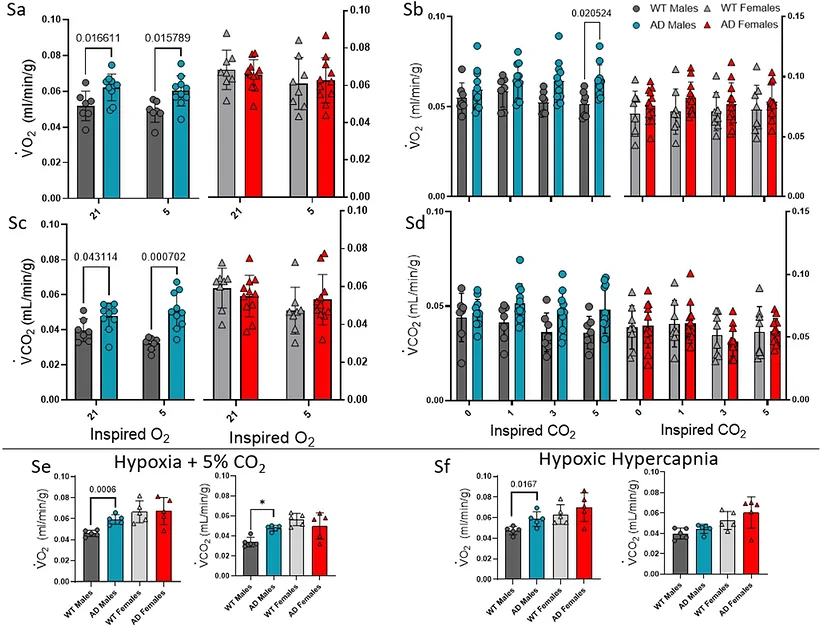
Metabolic responses to hypoxia and hypercapnia in 5XFAD mice Changes in ${\dot{V}}_{O_{2}}$ in hypoxic (Sa), hypercapnic (Sb), hypoxic + 5% CO_2_ (Se) and hypoxia hypercapnia (Sf) in WT and 5XFAD mice. Changes in ${\dot{V}}_{CO_{2}}$ in hypoxic (Sc), hypercapnic (Sd), hypoxic (10%) + 5% CO_2_ (Se), and hypoxia hypercapnia (5% CO_2_/8% O_2_) (Sf) in WT and 5XFAD mice. Error bars represent SD, and asterisks denote significant differences between WT and AD groups in males and females (significant *P*‐values are noted in the figure, *P* < 0.05). Multiple Student's *t* tests with Holm–Šídák *post hoc* test (*P* < 0.05); *n* = 7 (WT male), 8 (WT female), 11 (5XFAD male), and 12 (5XFAD female).

Since 5XFAD mice had increased apnoeas during presumptive sleep, we assessed their HVR (Fig. 2). We acutely challenged male and female WT and 5XFAD mice with 5% O_2_ and assessed the ability of 5XFAD mice to increase their ventilation (${\dot{V}}_{E}$), tidal volume (*V*_T_), frequency (*F*_R_) and ACR relative to their WT counterparts. In male 5XFAD mice, ${\dot{V}}_{E}$ (Fig. 2*A*,), *F*_R_ (Fig. 2*B*) and ACR (Fig. 2*D*), but not *V*_T_ (Fig. 2*C*) were blunted. In female 5XFAD mice, we found all measures of the HVR to be significantly impaired compared to WT females (Fig. 2*A*–*D*). Further, while the addition of CO_2_ to the 5% hypoxia (H+5) challenge expectedly increased all relative HVR measures in both genotypes and sexes (Fig. 2*A*–*D*), male 5XFAD mice had blunted capacities to increase their *V*_E_ (Fig. 2*A*), *V*_T_ (Fig. 2*C*) and ACR (Fig. 2*D*); *F*_R_ between male 5XFAD and WT mice did not differ (Fig. 2*B*). Female 5XFAD mice exhibited normal ${\dot{V}}_{E}$ and *F*_R_ responses to H+5 challenge but displayed blunted *V*_T_ (Fig. 2*C*) and ACR (Fig. 2*D*) responses. Little was observed in ${\dot{V}}_{O_{2}}$ and ${\dot{V}}_{CO_{2}}$ responses to acute hypoxia (Fig. A1).

**Figure 3:**
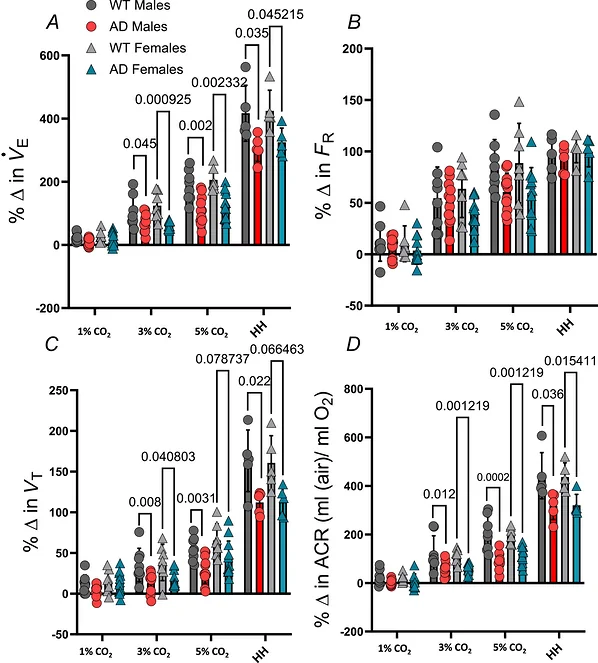
5XFAD mice have impaired ventilatory responses to hypercapnia Changes in: *A*, ventilation (${\dot{V}}_{E}$); *B*, breathing frequency (*F*_R_); *C*, tidal volume (*V*_T_); and *D*, the air convection requirement (ACR) to acute 5% hypercapnia were assessed in wild type (WT) and 5XFAD (AD) male and female mice. HH denotes hypoxic hypercapnia (5% CO_2_/8% O_2_) used to elicit a maximal ventilatory response. Error bars represent SD, and significant *P*‐values are noted in the figure (*P* < 0.05). Multiple Student's *t* tests with Holm–Šídák *post hoc* test; *n* = 7 (WT male), 8 (WT female), 11 (5XFAD male), and 12 (5XFAD female).

Similar to the HVR response, we found that both male and female 5XFAD mice had dampened HCVR (Fig. 3) measured following stepwise increases in inspired CO_2_ (from 1 to 5%). In all cases, 3% and 5% CO_2_ produced a significantly larger ventilatory response in WT animals compared to 5XFAD animals [*P* = 0.002, 0.008 (male) and 0.04, 0.002 (female) respectively]. *F*_R_ responses to hypercapnia were unaffected by the 5XFAD genotype, and thus the deficits in ${\dot{V}}_{E}$ were a consequence of impaired *V*_T_ (Fig. 3*B*, *C*). In both male and female 5XFAD animals, the ACR increased by ∼100% in 5% CO_2_, whereas it increased by ∼200% in WT animals (Fig. 3*D*; *P* < 0.03). Although challenge with hypoxic hypercapnia (HH; 5% CO_2_/8% O_2_) to elicit the maximum ventilatory response resulted in an increase in all measured ventilatory variables in both genotypes and sexes (Fig. 2*A*–*D*), 5XFAD males and females maintained a significantly blunted ventilatory response (Fig. 3*A*, *C*, *D*; *P* = 0.022 and 0.034 respectively), apart from *F*_R_. Little was observed in ${\dot{V}}_{O_{2}}$ and ${\dot{V}}_{CO_{2}}$ responses to hypercapnia (Fig. A1).

### Aβ plaque deposition occurs in brainstem regions associated with central chemoreception and correlates with impairments in the HCVR

**Figure 4:**
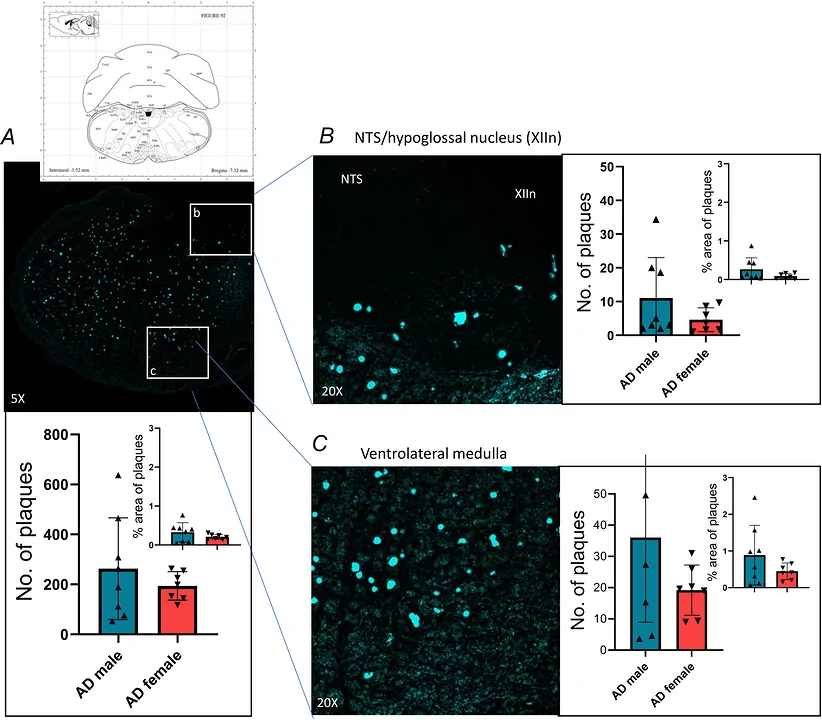
Representative images of Aβ plaques deposited in the medulla (*A*), the regions containing the NTS/hypoglossal nucleus (*B*) and VLM (*C*) as well as the number of Aβ plaques deposited in each region and the percentage area covered by plaques in male and female 5XFAD mice Plaques were stained using Methoxy‐X04; *n* = 8 (male) and 7 (female).

To identify a potential relationship between Aβ plaque load and respiratory dysfunction, we examined methoxy‐X04 staining in central brainstem medullary and pons regions responsible for chemosensory function and respiratory output. Total Aβ plaques were observed over 60 µm of the medulla in both male and female 5XFAD mice, and no sex differences were detected (Fig. 4*A*; males 268 ± 106.4, females 194.0 ± 21.6). Likewise, no sex differences were detected in the number of Aβ plaques in the hypoglossal motor nucleus (Fig. 4*B*; males 12.2 ± 5.66, females 4.62 ± 1.34) or the VLM, which contains respiratory rhythm‐generating sites (Fig. 4*C*; males 32.0 ± 12.74, females had 19.2 ± 3.04).

**Figure 5:**
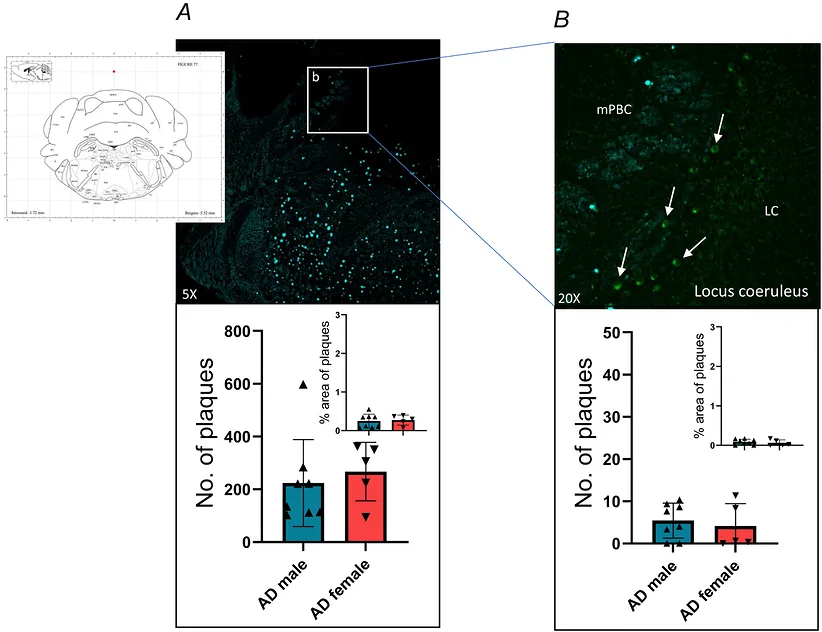
Representative images of Aβ plaques deposited in the pons (*A*), the region containing the locus coeruleus (LC) (*B*) as well as the number of Aβ plaques deposited and the percentage area covered by plaques in each region in male and female 5XFAD mice Plaques were stained using Methoxy‐X04. LC was stained with tyrosine hydroxylase; *n* = 8 (male) and 6 (female).

There were also no sex differences detected in the pontine region of the brainstem. 5XFAD males had on average 212 ± 86.2 plaques while 5XFAD females had 267.4 ± 44.4 plaques (Fig. 5*A*). Surprisingly, the LC consistently had very few plaques (males 4.0 ± 1.72, females 4.2 ± 2.2) compared to other medullary or pontine regions, and in many cases, no plaques were detected at all in the LC region (Fig. 5*B*). The percentage area covered by plaques in all pontine regions showed similar trends, suggesting no differences in plaque size (Fig. 5*A*,*B*). Also, as expected, no Aβ plaques were observed in WT animals (data not shown).

**Figure 6:**
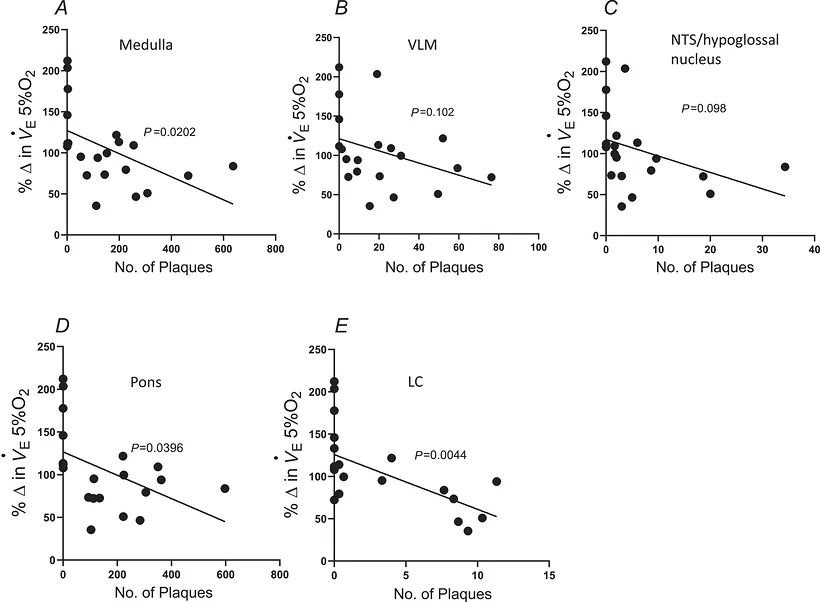
The relationship between the number of Aβ plaques and the increase in ventilation to acute 5% hypoxia in the medulla (*A*), the regions containing the VLM (*B*), NTS/hypoglossal nucleus (*C*), pons (*D*) and LC (*E*) An asterisk denotes whether the slope of the line is significantly different from 0 (simple linear regression), as well as a significant correlation (Pearson correlation) (significant *P*‐values are noted in the figure, *P* < 0.05); *n* = 7 (5XFAD male), 5 (5XFAD female), 3 (WT male), and 4 (WT female).

**Figure 7:**
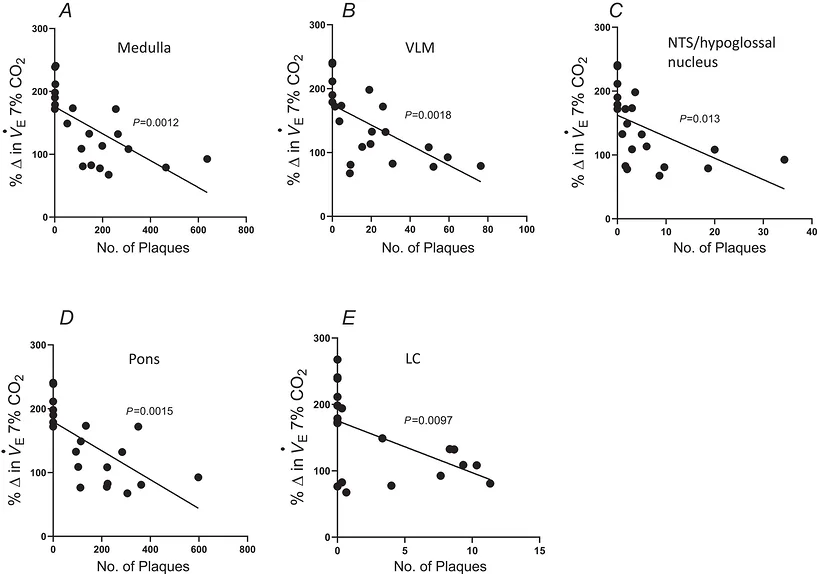
The relationship between the number of Aβ plaques and the increase in ventilation to 5% hypercapnia in the medulla (*A*), the regions containing the VLM (*B*), NTS/hypoglossal nucleus (*C*), pons (*D*) and locus coeruleus (LC) (*E*) An asterisk denotes whether the slope of the line is significantly different from 0 (simple linear regression), as well as a significant correlation (Pearson correlation) (significant *P*‐values are noted in the figure, *P* < 0.05); *n* = 7 (5XFAD male), 5 (5XFAD female), 3 (WT male) and 4 (WT female).

Aβ plaque deposition appeared to correlate primarily with changes in the HVR (Fig. 6). There was a significant correlation between the number of plaques and the percentage change in ventilation in response to 5% inspired O_2_ in the medulla (Fig. 6*A*), VLM (Fig. 6*B*), pons (Fig. 6*D*) and LC (Fig. 6*E*). We saw a negative correlation between the number of plaques and the percentage change in ventilation in response to 5% inspired O_2_ in the NTS/hypoglossal, but this did not reach statistical significance. Also of note, there was a strong negative correlation between Aβ plaque number and the HCVR (Fig. 7). The percentage change in ventilation in response to challenge with 5% CO_2_ was significantly correlated with all regions tested in the medulla (Fig. 7*A*), VLM (Fig. 7*B*), NTS/hypoglossal (Fig. 7*C*), pons (Fig. 7*D*) and LC (Fig. 7*E*)..

## Discussion

The goal of the present study was to better understand the extent to which respiratory disturbances observed in a mouse model of AD are associated with beta amyloid deposition in central chemoreceptive regions. We utilized the 5XFAD mouse model which develops beta amyloid plaques early in disease progression to help inform on this question. Previous work provides a strong framework correlating the occurrence of hypoxia with the emergence and progression of dementia, and ultimately AD (Dakterzada et al., 2025; Daulatzai, 2013; Gaeta et al., 2020; Gaig & Iranzo, 2012; Lee et al., 2019; Ogbu et al., 2024; Ulland et al., 2021). Indeed, hypoxia caused by SDB appears to not only be a risk factor for the development of AD but it also aggravates pathogenesis (Daulatzai, 2013; Leng et al., 2017; Marciante et al., 2022; Simic et al., 2009; Wrzesień et al., 2024; Yaffe et al., 2012). Thus, the cause of OSA and CSA in the context of AD is an intriguing and important question. Chemosensory feedback failure contributes to OSA and CSA, highlighting the criticality of the chemosensory feedback loop, which is perhaps the most vital system in maintaining homeostatic $P_{aO_{2}}$ and $P_{CO_{2}}$. Thus, understanding the nature of chemosensory dysfunction in amyloidosis and tauopathy is an important endeavour. Our data show that at 8 months of age, 5XFAD mice, a model of early amyloidosis, exhibit (1) a nearly 3‐fole increase in apnoeas during presumptive sleep, (2) deficient hypercapnic and hypoxic ventilatory responses, (3) Aβ plaque deposition in at least two regions associated with rhythm generation and central chemoreception, and (4) correlation between Aβ plaque load and an impaired hypercapnic ventilatory response. The implications of these findings are discussed below.

### Sleep‐disordered breathing and Alzheimer's disease

Considerable data connect SDB to neurodegenerative and neurological disorders (Daulatzai, 2013; Gaeta et al., 2020; Ogbu et al., 2024; Ulland et al., 2021). The present study describes the occurrence of apnoeas in the 5XFAD mouse model of amyloidosis, a control of breathing pathology also described in a murine tauopathy model of AD (Marciante et al., 2023). 5XFAD mice showed a notable increase in total apnoeas during presumptive sleep, having nearly triple the number of events compared to WT animals; spontaneous apnoeas accounted for most of this increase. In humans, the increase in the apnoea hypopnea index (AHI) in severe SDB (30 or more events per hour) can be 6–10 times higher than in healthy patients (Harvard Division of Sleep Medicine). In our study, the duration of spontaneous apnoea events in both WT and 5XFAD animals was similar. While the number of post‐sigh apnoeas did not differ between the two genotypes, their epoch duration was substantially longer (∼2 s) in 5XFAD animals. Previous studies have indicated that a difference in post‐sigh apnoea duration may significantly increase the degree of hypoxia experienced (Dempsey, 2019; Orr et al., 2017). Although it is possible that both obstructive and central apnoeas can occur in mice (Bartolucci et al., 2021; Dempsey, 2019; Leung et al., 2012; Orr et al., 2017), we suggest that central apnoeas are the more likely type given the rodent model used here; we did not attempt to distinguish them in the present study.

Hypoxia‐induced exacerbation of Alzheimer's pathology progression (Kazim et al., 2021; Marciante et al., 2022; Shiota et al., 2013; Sun et al., 2006; Ulland et al., 2021) suggests significant deleterious effects of SDB. Therefore, the degree to which the 5XFAD animals in the present study experienced hypoxia probably contributed to their worsened respiratory pathology. Further, administration of hypoxia consistently worsens AD‐like progression in all models tested. Even prenatal administration of hypoxia, designed to mimic high‐altitude environments, resulted in worsened AD‐like pathogenesis once the animals reached adulthood (Zhang et al., 2013). Hypoxia as an environmental risk factor for AD has also been posited, and SDB was at the forefront of the mechanistic basis for repeated hypoxic exposure (Daulatzai, 2013; Yaffe et al., 2012). Our observations that spontaneous apnoeas are already present at 8 months of age in the 5XFAD mouse model supports this idea. The contribution of hypoxia to the deposition of Aβ plaques and neurodegeneration in the context of human AD is therefore of great importance.

While the effect of hypoxia on increased cerebral amyloidosis and the occurrence of tau phosphorylation has been described in multiple AD models, causal mechanisms remain poorly understood. Hypoxia generally occurs via pauses in breathing (apnoeas) or due to tissue hypoperfusion as occurs with cerebral infarction or stroke (Borenstein et al., 2005; Li et al., 2009). Indeed, vascular dysfunction and SDB are often coincident. The deposition of Aβ is generated by the sequential cleavage of amyloid precursor protein (APP) by β‐ and γ‐secretases (Li et al., 2009), whose activities are positively regulated by hypoxia inducible factor 1 (HIF‐1), the primary molecule regulating oxygen homeostasis (Huang et al., 1999; Lahiri & Forster, 2003; Li et al., 2009; Sun et al., 2006). Further, β‐site β‐APP cleavage enzyme 1 (BACE1) is also upregulated in sporadic AD patients and by hypoxia, providing another link between hypoxia and Aβ plaques (Sun et al., 2006).

### Chemosensory feedback and sleep‐disordered breathing

The causes of OSA pathology involve, but are not limited to, genetic predispositions to reduced airway patency, obesity and craniofacial anatomy (Strollo & Rogers, 1996). Recently, the role of CSA in the occurrence of apnoeas during sleep and in OSA was examined (Orr et al., 2017). CSA results from insufficient drive from the central rhythm‐generating sites, an effect that is rooted primarily in dysfunctional chemosensory feedback (Dempsey, 2019; Orr et al., 2017). Paradoxically, this dysfunction is typically a consequence of hypersensitivity to hypercapnia and hypoxia rather than a reduced chemoresponse (Orr et al., 2017). This is thought to be the result of high‐loop gain where periodic overshoots and undershoots of homeostatic $P_{\mathrm{aC}O_{2}}$ result in nadirs in ventilatory drive such as occurs in Cheynne–Stokes breathing observed at altitude (Dempsey, 2019; Leung et al., 2012). In the present study, reduced chemosensory responses suggest a low‐loop gain situation where chemosensors fail to adequately respond to changes in blood gases, rather than high‐loop gain (Dempsey, 2019). While sparse reports exist, available data show that reduced ventilatory drive and chemoresponses can result in prolonged apnoeas and an elevated CO_2_ wake threshold (Gold et al., 1993; Orr et al., 2017). This is exemplified by elevated $P_{CO_{2}}$ levels and decreased ventilatory responses in reports of obese patients with sleep apnoea (Gold et al., 1993). Further, patients with neuromuscular disease and low‐loop gain show increased occurrences of prolonged apnoeas, highlighting the interaction of reduced ventilatory drive and muscular response to central output (reviewed in Orr et al., 2017). The nature of the apnoeas resulting from low‐loop gain in the present study and other murine models of AD is unclear (Brown et al., 2019; Marciante et al., 2022, 2023), so this interesting question remains unanswered.

There are multiple reports of reduced ventilatory response to hypercapnia in patients with SDB (Benarroch et al., 2007; Brown et al., 2019; Gold et al., 1993). In central congenital hypoventilation syndrome, central apnoeas result from reduced chemoresponses, and thus low‐loop gain (Orr et al., 2017). However, in most of these reports, patients are also challenged with co‐morbidities such as obesity. Not included in these co‐morbidities is AD. It is clear, however, that the most common cause of CSA is high‐loop gain, particularly in circumstances more closely related to the relationship between SDB and AD. This does beg the question of why the murine 5XFAD model has a reduction in sensitivity and an increase in spontaneous CSAs that are correlated with Aβ deposition.

Several rodent models of beta amyloidosis and tau hyperphosphorylation have been examined for changes in chemosensitivity, which may offer insight into the role of these pathologies in respiratory dysfunction. In the STZ model of AD in rats, extraordinary deposition of Aβ in the LC appears to contribute to the altered chemoresponse, presumably via increased inhibition of LC neurons to CO_2_ (Vicente et al., 2020). Interestingly, the STZ model also produced a decrease in the hypoxic ventilatory response (Brown et al., 2019), similar to our results. The TgF344‐AD transgenic rat model produced no change in ventilatory responses (Lucking et al., 2019) and the murine AβPP V717I model produced a high‐loop gain result (Andrzejewski et al., 2022). Tauopathy has been shown to notably decrease hypercapnic and hypoxic responses in mice (Marciante et al., 2023). In other models of amyloidosis (TgF344‐AD rats and AβPP V717I mice), the degree of amyloidosis and age probably affect the net effect on ventilatory responses. In the TgF344‐AD rats, Aβ deposition was not observed in the brainstem, though precursor c‐terminal fragments were detected (Lucking et al., 2019). In AβPP V717I mice, amyloid deposition was not reported in respiratory centres (Andrzejewski et al., 2022). Thus, it is very likely that progressive amyloidosis does not have a linear effect on chemosensory responses. The same may be true for tauopathy, and therefore the temporal nature of pathological progression on chemosensory responses is probably key to elucidating this question.

It is likely in our study that the SDB phenotype observed in 5XFAD mice results, in part, from chemosensory dysfunction (i.e. low‐loop gain), but a clear link has yet to be provided. There is, however, some evidence of this in the present study where no difference in post‐sigh activity in 5XFAD mice was observed. Increases in spontaneous apnoeas should result in some level of hypoxia, which is known to increase post‐sigh activity (Nakamura et al., 2003); however, we did not observe this, which could be the result of a decrease in the HVR. Examination of blood gases is critical to understanding this, and it is possible that with blunting of the HVR and HCVR, 5XFAD animals experienced even greater hypercapnia and hypoxia. If this were the case, 5XFAD mice would display an even more blunted HVR and HCVR given that they would have had stronger stimuli compared to WT animals.

### Aβ plaque disposition and chemosensory dysfunction

The distribution of Aβ plaques and tau pathology is well described in AD, but most examinations focus on CNS regions other than the brainstem. Several investigations into the occurrence of Aβ plaques and tauopathy in brainstem structures in humans have led to the focus on the LC and its contribution to cognitive decline and behavioural changes (Andrés‐Benito et al., 2017; Arendt et al., 2015; Iseki et al., 1989 *b*; Serra et al., 2018)

However, very little data exist regarding the effect of AD progression on other respiratory related regions in the brainstem, despite the overwhelming data supporting respiratory dysfunction in AD patients. In Tau.P301L mice, tauopathy in pontine and medullary brainstem regions has been described, and the Kolliker‐fuse, raphe obscurus and raphe magnus were found to be severely affected (Menuet et al., 2011). Further, synaptic loss in the nucleus tractus solitarius (NTS), a major integrative site for peripheral and central chemoreception, was observed to have pronounced synaptic loss in the rat STZ model of AD (Humphrey et al., 2023). 5‐HT neurons directly associated with respiratory function were found to be mostly unaffected, even though respiratory instability was still observed (Menuet et al., 2011).

In the present study, 5XFAD mice showed a substantial presence of Aβ plaques in the pons and medulla, with the VLM being consistently affected. The VLM in the present study probably contained part of the ventral respiratory column based on the presence of the hypoglossal nucleus in the same slice (Del Negro et al., 2018; Feldman et al., 2003; Ruangkittisakul et al., 2014). Comparatively, the NTS and hypoglossal nucleus, sites involved in respiratory integration and motor output (Baker‐Herman & Strey, 2011; Parisian et al., 2004), had far fewer Aβ plaques than the VLM, and in many cases, no plaques were detected. Similarly, while the pons did contain numerous Aβ plaques, the LC (a central chemoreceptor) rarely had plaques at this age (Gargaglioni et al., 2010). In fact, most plaques found in the region of the LC analysed were contained in the medial parabrachial complex (mPBC), just lateral to the LC. It has yet to be determined if aged 5XFAD mice show signs of neuronal loss in the LC at this stage (Flores‐Aguilar et al., 2022). Together, these data suggest that the chemosensory deficits observed were not due to plaque deposition in the LC, but rather, more likely due to plaques in the VLM (and thus central pattern generation), or perhaps another central chemoreceptor that is, as yet, unexamined.

Data from the present study do offer a glimpse into neuronal subtypes that are disproportionately affected by Aβ deposition. The LC is composed primarily of noradrenergic neurons where little amyloid deposition was observed, while the mPBC, a primarily glutamatergic site, had noticeably more Aβ plaques (Fig. 5). Other sites examined in the present study are more heterogenous in neuronal subtype composition. However, these observations only offer some insight. Important and interesting questions remain: notably, does Aβ plaque deposition exhibit disproportional neuron subtype selectivity (a question that our data only hint at being the case), and are there differences in function among the neuronal subtypes with Aβ deposition? While differences between brainstem regions were observed in this study, other regions may still be of interest based on our results. For example, the spinal vestibular nucleus (SpVe) region consistently was devoid of plaque deposition (like the LC) while more lateral regions like the parvicellular reticular nucleus alpha (PCRtA) were heavily impacted. There probably exists then (1) region or neuron specificity to plaque deposition, and (2) a reason for it. Understanding this relationship may be key in identifying the impact of Aβ deposition on brainstem function.

Whether the deficient chemosensory response we observe in 5XFAD mice is the result of direct effects on other central chemoreceptors located in the VLM, like the retrotrapezoid nucleus, or if there is dysfunction in the central rhythm generators themselves remains unknown. There appears to be a causal link between tauopathy and chemosensory and upper airway function (Dutschmann et al., 2010; Menuet et al., 2011). However, in our amyloid model both the HVR and the HCVR were reduced, so the relative location along the feedback loop where the deficits are rooted cannot be pinpointed. Hypoxia is sensed primarily at the carotid bodies (CBs) whereas hypercapnia is sensed both centrally (at multiple sites) and at the CBs (Guyenet et al., 2008; Lahiri & Forster, 2003; Nattie & Li, 2009; Nurse, 2014). Further, the deficits observed in the present study could also be the result of dysfunction not only at the chemoreceptor level, but also along the efferent pathways to the central pattern generators from the chemosensors, in addition to deficits in motor output itself (Hartzler & Putnam, 2009). However, the maximal ventilatory response to HH indicates that the deficits reported here cannot be due solely to mechanical impairments of the respiratory system or motor output since AD animals were able to further increase their respiratory effort with challenge, even if it was not to the same magnitude as in WT mice. This suggests that the deficits in the HVR are not due to the hypoxic response per se.

Aβ plaque deposition and tauopathy cause axonal and dendritic field changes that directly lead to neurite dystrophy, synaptic loss and eventually loss of neuronal viability (reviewed in Klucken et al., 2003; Spires & Hyman, 2004). In APP/PS1 mice, noradrenergic neurons in the LC have fewer tyrosine‐hydroxylase positive fibres and are shorter, thicker and broken (Liu et al., 2013). While neuronal viability was not examined in the present study, available data suggest the presence of Aβ plaques probably alter their function and morphology (Palop & Mucke, 2010). Further, neuroinflammation, a hallmark of AD pathology, also probably contributes to the chemosensory deficits observed in the present study (Ulland et al., 2021) though we did not quantify this. The contributions of neuroinflammation and microglial involvement in the respiratory dysfunction observed here in 5XFAD mice remain important questions (Marino et al., 2025; Ulland et al., 2021).

## Conclusion

Our data suggest that reductions in chemosensory feedback contribute to the increased apnoea phenotype in 5XFAD mice, a model of beta amyloid centric AD pathology in humans. Chemosensory feedback and its importance to maintaining a patent airway probably contributes to the intermittent hypoxia associated with SDB in AD patients. Chemosensor failure early in disease pathology in the 5XFAD model implicates amyloidosis as a risk factor and contributor to AD progression, though there are conflicting reports highlighting a need to further elucidate the link between amyloidosis and chemosensory dysfunction. We report that the 5XFAD mouse model has low‐loop gain in chemosensory drive and increases in apnoea incidence at 8 months of age. It is known that in murine models, hypoxia exacerbates tauopathy and amyloidosis (Marciante et al., 2022; Shiota et al., 2013) and it is suspected that hypoxia exacerbates human AD pathogenesis as well. CSA typically results from high‐loop gain, and enhanced sensitivity, and thus the nature of the change in chemosensitivity in different AD models remains an intriguing question given that studies have reported that amyloidosis gives rise to high‐loop gain as well as no effect, in addition to our own observation of low‐loop gain. Further, tau phosphorylation typically results in low‐loop gain, although that pathology is absent in the 5XFAD model. Untangling the independent and combined effects of amyloidosis and tauopathy on chemosensory dysfunction remains an important endeavour in understanding human AD pathology. While we report little difference in the metabolic response to hypoxia, it should be made clear that this study examined the ventilatory phase of the hypoxic response, and thus a metabolic response was not recorded. It may be interesting to examine the hypoxic metabolic response further, but mice respond to hypoxia very differently to humans. Still, understanding the metabolic adjustment to chronic hypoxia events in humans with AD might be useful. Further, understanding the temporal and mechanistic relationship between chemosensory failure, SDB incidence and AD pathogenesis will shed light on the role of hypoxia in AD regarding the proverbial chicken and egg. Additionally, the effect of Aβ plaque deposition and tauopathy on respiratory related regions, where the chemosensory deficits are rooted, in what stages of sleep the apnoeas occur, and if sleep architecture differs between WT and 5XFAD animals all remain open and important considerations for further study.

### Ethical approval

All methods were carried out in accordance with relevant local and University of Wisconsin guidelines and regulations. All animals were handled in accordance with the Animal Research: Reporting of *in vivo* Experiments (ARRIVE) guidelines and the University of Wisconsin's Institutional Animal Care and Use Committee policies and our approved protocols.

## Additional information

## Competing interests

The authors declare that they have no known competing financial interests or personal relationships that could have appeared to influence the work reported in this paper.

## Author contributions

R.J.S.: Conceptualization, Investigation, Data curation, Formal analysis, Methodology, Writing – original draft, Writing – review & editing. A.C.E.: Investigation, Data curation, Formal analysis. P.Z.R.: Data curation and Formal analysis. K.M.M.: Data curation and Formal analysis. M.G.G.: Data curation and Formal analysis. E.C.M.: Data curation and Formal analysis. A.B.R.: Data curation and formal analysis. T.K. Ulland: Conceptualization, Writing – original draft, Writing – review & editing, Supervision, and Funding Acquisition. J.J.W.: Conceptualization, Writing – original draft, Writing – review & editing, Supervision, and Funding Acquisition. T.L.B.: Conceptualization, Writing – original draft, Writing‐review & editing, Supervision, and Funding Acquisition.

## Funding

This work was supported by the National Institutes of Health T32AG000213 supporting RJS, R01HL142752‐03S1 to TKU, JJW, and TLB, R01HL142752 to JJW and TLB, R01AG070973 to TKU, R01AG083883 to TKU, P30AG062715 supporting TKU, and RF1AG082215 supporting TKU and KMM. Additional funding supporting this work includes the William F. Vilas Trust Estate Early‐Career Investigator Award to TKU, NSF grant DGE‐1 747 503 MGG, and the Herman H. Shapiro Wisconsin Distinguished Graduate Fellowship in Medicine from the University of Wisconsin Foundation to KMM.

## Generative AI statement

No generative artificial intelligence tools (GAI) were used in any part of this study.

## Supporting information


Peer Review History

## Data Availability

Data from this paper are available on request from the corresponding author (RJS).

## Appendix

**Table A1:** Average resting metabolic and ventilatory values.

	${\dot{V}}_{O_{2}}$ (ml/min/kg) (oxygen consumption rate)	*V*_T_ (ml/min/kg) (Tidal Volume)	*fR* (breath/min) (Breathing Freqency)	*V*_E_ (ml/min/kg) (Minute Ventilation)	ACR (Air Convection Requirement, VE/VO2)	*T*_b_ (°C) (Body Temperature)	Sighs (/h)	Sleep duration (h)
WT male	0.055 ± 0.007	0.271 ± 0.036	145.04 ± 21.41	0.96 ± 0.073	17.11 ± 2.84	36.16 ± 0.55	12.90 ± 3.85	0.808 ± 0.14
AD male	0.059 ± 0.011	0.239 ± 0.037	150.59 ± 24.06	1.06 ± 0.22	18.08 ± 3.31	36.44 ± 0.56	14.70 ± 4.50	0.88 ± 0.11
WT female	0.070 ± 0.017	0.235 ± 0.040	132.37 ± 20.13	1.04 ± 0.11	16.16 ± 3.41	36.39 ± 0.712	13.90 ± 1.97	0.79 ± 0.11
AD female	0.076 ± 0.013	0.246 ± 0.045	147.26 ± 17.04	1.35 ± 0.19	18.14 ± 3.17	36.55 ± 0.75	14.70 ± 2.68	0.79 ± 0.10

Data are presented as ± SD. Number of sighs per hour was determined during presumptive sleep. *n* = 7 (WT male), 8 (WT female), 11 (5XFAD male), and 12 (5XFAD female).
