# Supplementary material for: Amyloid beta plaque pathology induces chemosensory deficits and increases apnoeas in the 5XFAD mouse model of Alzheimer's disease
Source: J Physiol. 2026 Sep 5;604(18):7971–89. doi: 10.1113/JP290936 (PMC13577700; doi:10.1113/JP290936)
Supplement: Supplementary file 1 [file TJP-604-7971-s001.pdf]

The Journal of Physiology

Alzheimer's disease: Amyloid beta plaque pathology induces chemosensory deficits and increases apneas in the 5XFAD mouse model of Alzheimer's disease

Ryan J Sprenger, Andrea C Ewald, Phinea Z Romero, Kaitlyn M Marino, Maia G Gumnit, Erin C McCann, Abigail B Radcliff, Tyler K Ulland, Jyoti Watters, and Tracy L Baker
DOI: 10.1113/JP290936

Corresponding author(s): Ryan Sprenger (rsprenger16@alumni.uwosh.edu)

The referees have opted to remain anonymous.

Review Timeline:

Submission Date:	14-Jan-2026
Editorial Decision:	24-Feb-2026
Revision Received:	24-Mar-2026
Editorial Decision:	20-Apr-2026
Revision Received:	26-May-2026
Editorial Decision:	17-Jun-2026
Revision Received:	23-Jun-2026
Accepted:	06-Aug-2026

Senior Editor: Bettina Mittendorfer

Reviewing Editor: Jing-Ning Zhu

Transaction Report:

(Note: With the exception of the correction of typographical or spelling errors that could be a source of ambiguity, letters and reports are not edited. Depending on transfer agreements, referee reports obtained elsewhere may or may not be included in this compilation. Referee reports are anonymous unless the Referee chooses to sign their reports.)

Dear Dr Sprenger,

Re: JP-RP-2026-290936 "**Alzheimer's disease: Amyloid beta plaque pathology induces chemosensory deficits and increases apneas in the 5XFAD mouse model of Alzheimer's disease** " by Ryan J Sprenger, Andrea C Ewald, Phinea Z Romero, Kaitlyn M Marino, Maia G Gumnit, Erin C McCann, Abigail B Radcliff, Tyler K Ulland, Jyoti Watters, and Tracy L Baker

Thank you for submitting your manuscript to The Journal of Physiology. It has been assessed by a Reviewing Editor and by 2 expert referees and we are pleased to tell you that it is acceptable for publication following satisfactory revision.

The referee reports are copied at the end of this email.

Please address all the points raised and incorporate all requested revisions or explain in your Response to Referees why a change has not been made. We hope you will find the comments helpful and that you will be able to return your revised manuscript within 4 weeks. If you require longer than this, please contact journal staff: jp@physoc.org.

Your revised manuscript should be submitted online using the link in your Author Tasks Link Not Available. This link is accessible via your account as Corresponding Author; it is not available to your co-authors. If this presents a problem, please contact journal staff (jp@physoc.org). Image files from the previous version are retained on the system. Please ensure you replace or remove any files that are being revised.

If you do not wish to submit a revised version of your manuscript, you must inform our journal staff (jp@physoc.org) or reply to this email to request withdrawal. Please note that a manuscript must be formally withdrawn from the peer review process at one journal before it may be submitted to another journal.

TRANSPARENT PEER REVIEW POLICY: To improve the transparency of its peer review process The Journal of Physiology publishes online, as supporting information, the peer review history of all articles accepted for publication. Readers will have access to decision letters, including Editors' comments and referee reports, for each version of the manuscript, as well as any author responses to peer review comments. Referees can decide whether or not they wish to be named on the peer review history document.

ABSTRACT FIGURES: Authors are expected to use The Journal's premium BioRender account to create/redraw their Abstract Figures. Information on how to access this account is here: https://physoc.onlinelibrary.wiley.com/journal/14697793/biorender-access.

This will enable Authors to create and download high-resolution figures. If authors have used the free BioRender service, they can use the instructions provided in the link above to download a high-resolution version suitable for publication.

The link provided should only be used for the purposes of this submission. Authors will be charged for figures created on this account if they are not related to this manuscript submission.

LANGUAGE EDITING AND SUPPORT FOR PUBLICATION: If you would like help with English language editing, or other article preparation support, Wiley Editing Services offers expert help, including English Language Editing, as well as translation, manuscript formatting, and figure formatting at www.wileyauthors.com/eeo/preparation. You can also find resources for Preparing Your Article for general guidance about writing and preparing your manuscript at www.wileyauthors.com/eeo/prepresources.

RIGOUR AND REPRODUCIBILITY: We recommend authors consult The Journal's Rigour and Reproducibility requirements to ensure that their manuscript is suitable for publication: https://physoc.onlinelibrary.wiley.com/pb-assets/hub-assets/physoc/documents/TJP-Rigour-and-Reproducibility-Requirements.pdf

REVISION CHECKLIST:

Check that your Methods section conforms to journal policy: https://jp.msubmit.net/cgi-bin/main.plex?form_type=display_requirements#methods

Check that data presented conforms to the statistics policy: https://jp.msubmit.net/cgi-bin/main.plex?form_type=display_requirements#statistics

Upload a full Response to Referees file. To create your 'Response to Referees' copy all the reports, including any comments from the Senior and Reviewing Editors, into a Microsoft Word, or similar, file and respond to each point, using font or background colour to distinguish comments and responses and upload as the required file type.

Please upload two versions of your manuscript text: one with all relevant changes highlighted and one clean version with no changes tracked. The manuscript file should include all tables and figure legends, but each figure/graph should be uploaded as separate, high-resolution files. The journal is now integrated with Wiley's Image Checking service. For further details,

see: https://www.wiley.com/en-us/network/publishing/research-publishing/trending-stories/upholding-image-integrity-wileys-image-screening-service

You may also upload:

- 'Potential Cover Art' for consideration as the issue's cover image
- Appropriate Supporting Information (Video, audio or data set: see https://jp.msubmit.net/cgi-bin/main.plex?form_type=display_requirements#supp).

We look forward to receiving your revised submission.

If you have any queries, please reply to this email and we will be pleased to advise.

Yours sincerely,

Bettina Mittendorfer
Senior Editor
The Journal of Physiology

REQUIRED ITEMS

- Author photo and profile. First or joint first authors are asked to provide a short biography (no more than 100 words for one author or 150 words in total for joint first authors) and a portrait photograph. These should be uploaded and clearly labelled together in a Word document with the revised version of the manuscript. See Information for Authors for further details.

- You must start the Methods section with a paragraph headed Ethical approval (https://jp.msubmit.net/cgi-bin/main.plex?form_type=display_requirements#methods).

Research must comply with The Journal's policies regarding animal experiments (https://physoc.onlinelibrary.wiley.com/hub/animal-experiments) and adherence to these policies must be stated in the manuscript.

Authors should confirm in their Methods section that their experiments were carried out according to the guidelines laid down by their institution's animal welfare committee, including an ethics approval reference number. The Methods section must contain a statement about access to food, water and housing, details of the anaesthetic regime: anaesthetic used, dose and route of administration, and method of killing the experimental animals.

- Please upload separate high-quality figure files via the submission form.

- Please ensure that any tables are editable and in Word format, and wherever possible, embedded in the article file itself.

- Please ensure that the Article File you upload is a Word file.

- Papers must comply with the Statistics Policy: https://jp.msubmit.net/cgi-bin/main.plex?form_type=display_requirements#statistics.

In summary:

- If n {less than or equal to} 30, all data points must be plotted in the figure in a way that reveals their range and distribution. A bar graph with data points overlaid, a box and whisker plot or a violin plot (preferably with data points included) are acceptable formats.

- If n > 30, then the entire raw dataset must be made available either as supporting information, or hosted on a not-for-profit repository, e.g. FigShare, with access details provided in the manuscript.

- 'n' clearly defined (e.g. x cells from y slices in z animals) in the Methods. Authors should be mindful of pseudoreplication.

- All relevant 'n' values must be clearly stated in the main text, figures and tables.

- The most appropriate summary statistic (e.g. mean or median and standard deviation) must be used. Standard Error of the Mean (SEM) alone is not permitted.

- Exact p values must be stated. Authors must not use 'greater than' or 'less than'. Exact p values must be stated to three significant figures even when 'no statistical significance' is claimed.

- Please include an Abstract Figure file and an Abstract Figure legend. An appropriate figure legend, which should not exceed 150 words in length, should be included in the main manuscript file. The Abstract Figure is a piece of artwork designed to give readers an immediate understanding of the research and should summarise the main conclusions. If possible, the image should be easily 'readable' from left to right or top to bottom. It should show the physiological relevance of the manuscript so readers can assess the importance and content of its findings. Abstract Figures should not merely recapitulate other figures in the manuscript. Please try to keep the diagram as simple as possible and without superfluous information that may distract from the main conclusion(s). Abstract Figures must be provided by authors no later than the revised manuscript stage and should be uploaded as a separate file during online submission labelled as File Type 'Abstract Figure'. Please also ensure that you include the figure legend in the main article file. All Abstract Figures should be created using BioRender. Authors should use The Journal's premium BioRender account to export high-resolution images. Details on how to use and access the premium account are included as part of this email.

EDITOR COMMENTS

Reviewing Editor:
Thank you for submitting your manuscript to the Journal of Physiology. We have now received comments from two expert reviewers. Both reviewers recognize the significance of your work in addressing breathing dysfunction in Alzheimer's disease models. However, several key concerns require revision, including model interpretation (the 5xFAD mouse should be framed as a model of beta-amyloidosis rather than directly equated to human AD), physiological interpretation (the relationship between chemoreflex function and apnea stability is mischaracterized in places, and terminology around chemoreflex testing, e.g., "isocapnic hypoxia", needs greater precision), and literature engagement (key relevant studies are not cited or discussed). Besides, further analyses would strengthen the study: analysis of which sleep stages (NREM vs. REM) the observed apneas occur in, whether sleep architecture differs between groups, and whether Abeta plaque deposition in brainstem respiratory neurons exhibits subtype specificity.

REFEREE COMMENTS

Referee #1:

Disordered breathing is common in many disease states and despite evidence that beathing problems negatively impact quality of life and contribute to disease progression, surprisingly little is known about underlying mechanisms. Therefore, I consider this studies effort to understand the basis of breathing problems in Alzheimer's disease (AD) to be highly significant. Their ability to use the respiratory waveform as a means of estimating sleep wake status is a clever alternative to more invasive approaches. They show that a mouse model of AD exhibits a profound apnea phenotype that likely results from diminished central and peripheral chemoreflex function. They also provide the first evidence that I'm aware of showing amyloid beta plaques in brainstem regions including those associated with control of breathing. Experiments are well controlled, carefully interpreted, and provide important insight regarding the relevance of breathing problems in AD. I have only minor suggestions (see point-by-point below).

1) What is the rationale for looking for sex differences? are breathing problems or mortality associated with AD sexually dimorphic?

2) I like that effort to distinguish spontaneous apnea from post-sigh apnea. Perhaps I missed it but its not clear to me how

apneic events are defined. Typically, 2 or more missed breaths until breathing resumes to near normal levels. Its important to make clear when the apnea is over because multiple breaths are required to recover blood gases. I point this out because its not clear if the two arrows in Fig. 1a (top) identify two apneic events (1 post-sigh apnea and 1 spontaneous apnea) interspersed by a single breathe or one long postsigh apnea? I think the later.

3) 5XFAD mice exhibit an apneic phenotype which is expected to result in some degree of hypoxia. Despite this, these animals show normal sigh activity. This is interesting because hypoxia strongly elicits sigh activity. Therefore, this observation is consistent with the possibility these mice have a reduced hypoxia sensitivity. This seems like an important point that should be emphasized.

4) Size of the extracellular space, and consequently protein clearance, is regulated across sleep wake states, so I wonder if 5XFAD mice showed any difference in time spent in presumed sleep?

5) The lack of plaques in the LC is interesting. Is this unique to the LC or are there other areas devoid of plaques or maybe hot spots?

6) Results show that 5XFAD mice have a blunted chemoreflex (central and peripheral) so why does the first sentence of the conclusion paragraph say 'augmented chemosensory feedback'?

7) Fig. 3 needs to define each symbol/color (like fig 2)

8) Figs 4-5 would benefit from including a atlas image.

9) To help readers outside the field appreciate the relevance of disordered breathing (specifically central chemoreception) in disease it would be nice to cite this review (PMID: 40816914).

Referee #2:

This paper presents interesting data demonstrating that the transgenic murine model of beta amyloidosis (5xFAD; a presumptive AD model) exhibits disturbances in the control of breathing including increased spontaneous apneas during presumptive sleep and diminished ventilatory responses to hypoxia or elevated carbon dioxide levels. The authors interpret their findings in the context of human Alzheimer's disease, suggesting that beta amyloid deposition contributes to breathing instability, which can in turn exacerbate disease progression. Although the data are interesting, and worthy of publication, there are major flaws in the presentation of the manuscript including over-interpretation of the murine model, misinterpretation of literature on central sleep apnea, and omission of several publications in the past two years very closely linked with this study.

Major concerns:

The manuscript writing should be improved for clarity and accuracy by being more precise in how things are phrased. There are many points where statements go well beyond any available evidence, or even contradict the very papers cited to buttress their arguments. Items of particular concern are:

• The 5xFAD mouse (Tg6799), an interesting model of beta-amyloidosis, does incorporate many human amyloid genetic variants linked to AD, but fails to consider other potential causes of AD pathogenesis, such as Tau variants. It is inappropriate to simply assume this mouse model reflects human AD. It is not inclusive and it is a mouse. Much effort has been spent developing drugs targeting beta-amyloid to treat human AD, but they have met with very limited success. This history alone suggests a beta amyloid mouse must be interpreted with care---it is a model of beta amyloidosis (worthy by itself). Whether it reflects AD is a topic for another day. Writing should reflect that caution.

• Other widely studied murine models of AD based on tau variants are available and data on breathing control (apneas and

chemoreflexes) show even greater chemoreflex impairment and spontaneous apneas (Marciante et al., 2023; PMID: 37811498). This study is not cited, but other rodent models with far less support as AD models are (streptozotocin). Another paper concerning beta amyloid over-expression and breathing with results contrary to the present study is ignored (Andrzejewski et al., 2022; PMID: 35682682). Yet another concerning beta amyloid in rats is not mentioned that reports contrary results (Lucking et al., 2019; PMID: 29969703). It took very little to find these papers in a search. These and any other relevant papers should be discussed.

• On the flip side, the authors discuss the idea that there is a positive feedback loop where sleep disordered breathing accelerates either beta amyloid deposition (and one could again add Tau phosphorylation). There are several highly relevant references for this argument that are not cited (found during the search above).

• In the abstract and introduction, the authors claim that suppressed chemoreflexes are associated with central apneas during sleep. The two papers cited to support that conclude that high, not low loop gain is responsible (Orr et al., 2017; Dempsey, 2019). This misleading or poorly worded writing undermines the manuscript. Although high loop gain is addressed in the discussion, evidence that low loop gain also causes unstable breathing relies on potentially unrelated/misleading cases such as CCHS. This flaw does not undermine the value of the study, but does undermine this manuscript.

• As per above, the authors say: "Chemosensory feedback failure contributes to OSA and CSA, yet the function of the chemosensory feedback loop, which is the most vital system in maintaining homeostatic PaO2 and PCO2 has not been linked to sleep apnea in AD." This statement is not actually correct. Further, nothing presented here directly links unstable breathing with an acceleration of pathology (plaque formation) or disease progression (neurodegeneration)-although the correlations presented are interesting.

• Statistics need further description and justification. Welch's t test does not correct for multiple comparisons. It is also unclear when it was used specifically. Similarly, it should be specifically stated when the ANOVA was used. This information could be added to each specific comparison presented in the results and/or figures; otherwise, it is hard to tell if they were applied in an appropriate manner. If Grubb's test was used to preclude data, how often were significant outliers found and excluded? Were other criteria included (such as observed sniffing or other behaviors)?

• For chemoreflex assessments, use of inspired gas alterations is common for mice, but it is not appropriate to say things like isocapnic hypoxia without measuring blood gases. Further, inspired carbon dioxide or oxygen are not the relevant stimuli and are used as a proxy because mice are so small. It is appropriate (and important) to consider translation from inspired to arterial gases, and potential confounds in the data set. For example, with a lower chemoreflex, CO2 levels go even higher and oxygen lower at the same inspired gas levels. What impact would this have? Also, is there any evidence whole mouse genetic manipulations affected pulmonary mechanics or gas exchange in a way that could alter the apparent stimulus/response curve?

Minor points:

• The first paragraph of the introduction leads readers in the wrong direction. This study is not about AD, it is about the impact of amyloidosis on breathing, which may be linked to AD. It is suggested to collapse this paragraph into one sentence and get on with the purpose of this study.

• During plethysmography, 4 hours without water or food is a long time for a small rodent. Was any provided?

• Were the inspired CO2 levels randomized or presented in sequence? The latter can raise some concerns.

• The statement is made that there is "profound" increase in sleep apnea. In the context of sleep apnea in humans, a doubling is not profound. Such words should be used sparingly. How does the apnea rate compare to a sleep apnea patient?

• Please state more clearly how an apnea was defined.

• Many details are not dealt with, such as dots over the VE and VO2 symbols.

• Why was VO2 reported and not VCO2, which is more closely linked with breathing? Please add a graph of VO2 in the different conditions. That is very important variable in a small mammal, and its regulation could be altered by beta amyloid in the wrong place.

• Were there any differences in temperature or metabolic responses to hypoxia between the strains?

• The chemoreflex blunting is important, yet not as profound as reported in a Tau model of AD. Is this potentially due to differences in tau vs amyloid, or age or other factors?

END OF COMMENTS

Responses to Editor and Reviewers

We would like to sincerely thank the editor and reviewers of this manuscript. The suggested changes have made for a better manuscript. We sought to adjust for every comment and suggestion with only a few that are logistically not feasible at this stage. We have noted this in our responses to each comment. Please see specific responses below in red.

Editor:

- analysis of which sleep stages (NREM vs. REM) the observed apneas occur in
  The authors of this manuscript agree that this would add value to the study, however all of the PIs and research personnel that conducted the study are retired or moved to other positions making this analysis logistically not feasible.
- whether sleep architecture differs between groups
  For the same reason as the first analysis suggestion, this analysis would be logistically not feasible.
- whether Abeta plaque deposition in brainstem respiratory neurons exhibits subtype specificity

  The authors agree that this is an interesting question. When we designed the study, we thought that noradrenergic cells of the LC would be disproportionately affected given their importance in sleep and chemoreception. However, our results suggested otherwise. Because of this, though, we only stained for noradrenergic LC neurons (much of the LC). We can infer from other sites that are composed primarily of one subtype whether there is subtype specificity, but we won't have the ability to re-stain. We have expanded this discussion to address this comment though in lines 414-427.

Reviewer 1:

- What is the rationale for looking for sex differences? are breathing problems or mortality associated with AD sexually dimorphic?
  - We included sex difference analysis for two main reasons, 1) women are disproportionately affected by AD nearly doubling men in diagnosis even after accounting for long-lifespans of women and 2) OSA contributes to dementia risk to a greater degree in women (https://doi.org/10.1093/sleepadvances/zpae077). We wanted to try to understand if amyloidosis deposition in the brainstem respiratory regions disproportionately affected female mice. We did not control however for estrus cycle.

- I like that effort to distinguish spontaneous apnea from post-sigh apnea. Perhaps I missed it but its not clear to me how apneic events are defined. Typically, 2 or more missed breaths until breathing resumes to near normal levels. Its important to make clear when the apnea is over because multiple breaths are required to recover blood gases. I point this out because its not clear if the two arrows in Fig. 1a (top) identify two apneic events (1 post-sigh apnea and 1 spontaneous apnea) interspersed by a single breathe or one long postsigh apnea? I think the later.
    - Thank you for catching this. We have updated the definitions of apneas in the methods lines (162-164). Yes, 2 or more skipped breaths for apneas. For post sigh apneas, we examined a 10s epoch after the sigh for apneas that fit the “2 skipped breaths” paradigm. For figure 1 a top, that is an example of two post sigh apneas with that 10s epoch.
- 5XFAD mice exhibit an apneic phenotype which is expected to result in some degree of hypoxia. Despite this, these animals show normal sigh activity. This is interesting because hypoxia strongly elicits sigh activity. Therefore, this observation is consistent with the possibility these mice have a reduced hypoxia sensitivity. This seems like an important point that should be emphasized.
    - This is a thoughtful point; we have added wording on this to the discussion lines (375-383)
- Size of the extracellular space, and consequently protein clearance, is regulated across sleep wake states, so I wonder if 5XFAD mice showed any difference in time spent in presumed sleep?
    - We have added these values to table 1. We did not see any notable difference in sleep duration, but this could very well be due to experimental design. It would be interesting to examine this question in an unrestrained “home cage” chronic analysis.
- The lack of plaques in the LC is interesting. Is this unique to the LC or are there other areas devoid of plaques or maybe hot spots?
    - We were surprised to not find much going on in the LC. We did check other respiratory regions including the NTS, hypoglossal nucleus and the VLM. Many of regions did have plaque deposition but none that stood out as a hot spot. Figure 4a really shows a nice distribution map where lateral parts of the brainstem seemed to be disproportionately affected. There are autonomic sites in that area that can have an influence on breathing (ex. **PCRtA).** One area that did stand out though consistently was just lateral to the NTS which would be the SpVe, this was typically devoid of plaques. But I think this is a question that should be examined in more detail than the scope of this study. The RVLM lands locationally similarly to the VLM and it would be interesting to know if this site is affected as well. We’ve added a short discussion of this in lines (414-427).

- Results show that 5XFAD mice have a blunted chemoreflex (central and peripheral) so why does the first sentence of the conclusion paragraph say 'augmented chemosensory feedback'?
  - A tenuous grasp on the English language apparently, fixed thank you. I found several other spots where this word was misused.
- Fig. 3 needs to define each symbol/color (like fig 2)
  - Fixed thank you
- Figs 4-5 would benefit from including a atlas image.
  - Agreed and added
- To help readers outside the field appreciate the relevance of disordered breathing (specifically central chemoreception) in disease it would be nice to cite this review (PMID: 40816914).
  - Added, thank you.

Reviewer 2:

- The 5xFAD mouse (Tg6799), an interesting model of beta-amyloidosis, does incorporate many human amyloid genetic variants linked to AD, but fails to consider other potential causes of AD pathogenesis, such as Tau variants. It is inappropriate to simply assume this mouse model reflects human AD. It is not inclusive and it is a mouse. Much effort has been spent developing drugs targeting beta-amyloid to treat human AD, but they have met with very limited success. This history alone suggests a beta amyloid mouse must be interpreted with care---it is a model of beta amyloidosis (worthy by itself). Whether it reflects AD is a topic for another day. Writing should reflect that caution.
  - We have gone through the manuscript to ensure more caution is had with this topic. Thank you for highlighting this.
- Other widely studied murine models of AD based on tau variants are available and data on breathing control (apneas and chemoreflexes) show even greater chemoreflex impairment and spontaneous apneas (Marciante et al., 2023; PMID: 37811498). This study is not cited, but other rodent models with far less support as AD models are (streptozotocin). Another paper concerning beta amyloid over-expression and breathing with results contrary to the present study is ignored (Andrzejewski et al., 2022; PMID: 35682682). Yet another concerning beta amyloid in rats is not mentioned that reports contrary results (Lucking et al., 2019; PMID: 29969703). It took very little to find these papers in a search. These and any other relevant papers should be discussed.
  - We have added a paragraph in the discussion to adequately cover the available models that have respiratory data. The original manuscript was

    - written before many of these papers and better care should have been taken to update it (lines 357-373).
- On the flip side, the authors discuss the idea that there is a positive feedback loop where sleep disordered breathing accelerates either beta amyloid deposition (and one could again add Tau phosphorylation). There are several highly relevant references for this argument that are not cited (found during the search above).
    - We have added these references to the discussion and conclusion.
- In the abstract and introduction, the authors claim that suppressed chemoreflexes are associated with central apneas during sleep. The two papers cited to support that conclude that high, not low loop gain is responsible (Orr et al., 2017; Dempsey, 2019). This misleading or poorly worded writing undermines the manuscript. Although high loop gain is addressed in the discussion, evidence that low loop gain also causes unstable breathing relies on potentially unrelated/misleading cases such as CCHS. This flaw does not undermine the value of the study, but does undermine this manuscript.
    - The manuscript has been revised to more carefully talk about loop gain. We agree that CSAs are most often the result of high loop gain while some evidence of low loop gain being the culprit in certain circumstances exist. With regard to this manuscript, the mistake was in poor wording choice describing a malfunctioning chemosensory feedback. Wording was made more clear that this malfunction in CSA is high loop gain and not loss of sensitivity. Pertaining to the discussion, we added a section clarifying this point and examining more of the available data on chemsensory drive in other amyloid and tau models (see second comment as well) to better describe the current mix of results and why some models produce decreases while other increases in sensitivity (lines 353-373).
- As per above, the authors say: "Chemosensory feedback failure contributes to OSA and CSA, yet the function of the chemosensory feedback loop, which is the most vital system in maintaining homeostatic PaO2 and PCO2 has not been linked to sleep apnea in AD." This statement is not actually correct. Further, nothing presented here directly links unstable breathing with an acceleration of pathology (plaque formation) or disease progression (neurodegeneration)- although the correlations presented are interesting
    - Phrasing on this has been corrected to be clearer that this question is being actively investigated in pre-clinical models. We also added one citation reporting dose-dependent tau-phosphorylation in hypoxia to better connect disease progression due to OSA/CSA. We agree the correlations are interesting and this has been clarified in the conclusion as well (lines 290-292, 467-469).

- Statistics need further description and justification. Welch's t test does not correct for multiple comparisons. It is also unclear when it was used specifically. Similarly, it should be specifically stated when the ANOVA was used. This information could be added to each specific comparison presented in the results and/or figures; otherwise, it is hard to tell if they were applied in an appropriate manner. If Grubb's test was used to preclude data, how often were significant outliers found and excluded? Were other criteria included (such as observed sniffing or other behaviors)?
    - Clarification on which tests were used and where were added to the methods and the figure legends. Welches were used on the sleep data given that only two groups existed for each test, meaning there weren't multiple comparisons being made. All other tests utilized the Holm-Šídák post hoc test for multiple comparison corrections, except for the correlation plots. We have clarified those stats as well.
    - We have made the appropriate correction on the outlier identification for sleep data. A Grubb's test was inappropriately utilized given it can identify only one outlier in a set of data, instead we used a ROUT test from Graphpad Prism allowing for multiple outliers to be identified. We used a Q=0.1 to identify definitive outliers. We did identify 2% of the apnea durations were identified as outliers using this method as well but chose not to exclude given that apneas are easily and definitively identified from the plethysmography data. This has been updated in the methods section.
- For chemoreflex assessments, use of inspired gas alterations is common for mice, but it is not appropriate to say things like isocapnic hypoxia without measuring blood gases. Further, inspired carbon dioxide or oxygen are not the relevant stimuli and are used as a proxy because mice are so small. It is appropriate (and important) to consider translation from inspired to arterial gases, and potential confounds in the data set. For example, with a lower chemoreflex, CO2 levels go even higher and oxygen lower at the same inspired gas levels. What impact would this have? Also, is there any evidence whole mouse genetic manipulations affected pulmonary mechanics or gas exchange in a way that could alter the apparent stimulus/response curve?
    - We have adjusted the usage of the word Isocapnic hypoxia to hypoxia+5%CO_2. The authors agree that without blood gases we cannot assess if the animals were isocapnic, though 5% CO_2 should get them quite close (I've seen 3.5-5% CO_2 used). It is a good point though that with blunting of the HCVR, 5% CO_2 will likely have resulted in differences in blood gases at the hypercapnia/hypoxia exposures. We have added this phrasing to the discussion (lines 378-381).

- It is difficult to assess if the genetic manipulation of this model per se affected mechanics or gas exchange. We are unaware of data showing pulmonary dysfunction in the 5XFAD model.

• The first paragraph of the introduction leads readers in the wrong direction. This study is not about AD, it is about the impact of amyloidosis on breathing, which may be linked to AD. It is suggested to collapse this paragraph into one sentence and get on with the purpose of this study.

We have collapsed this to two sentences to set up aging as a risk then move on to the rest of the introduction.

• During plethysmography, 4 hours without water or food is a long time for a small rodent. Was any provided?

Neither food nor water were provided. It is long, but this was required to allow animals time enough to sleep. Animals spent just under an hour of this period sleeping, typically.

• Were the inspired CO2 levels randomized or presented in sequence? The latter can raise some concerns.

- In the present study, we exposed sequentially without returns to air in between. This was to reduce time in the chamber. Many studies do use a sequential method, however we recognize the concerns with this method primarily with STP and order bias. This would make these data harder to compare to non-sequential data, but still valuable for comparison between our groups given the use of the same method between the 5XFAD and WT animals. This has been clarified in the methods for readers.

- The statement is made that there is "profound" increase in sleep apnea. In the context of sleep apnea in humans, a doubling is not profound. Such words should be used sparingly. How does the apnea rate compare to a sleep apnea patient?

This wording has been softened. While we see nearly triple the number of apneas, SDB patients with mild pathology have ~2-3x, moderate ~3-6X and severe more than 6X the number of apneas. That direct translation from mouse to human might be tricky though. The real comparison is how often and to what degree is hypoxia/hypercapnia happening given an increase in apneas. The effect of 1 apnea in humans likely doesn't equal 1 apnea in a mouse. It is also key to remember that mice handle hypoxia differently than humans. The best comparison for a study like this is within the species (lines 299-301).

• Please state more clearly how an apnea was defined.

Added in the methods section, thank you for identifying this

• Many details are not dealt with, such as dots over the VE and VO2 symbols. These have been added where appropriate

• Why was VO2 reported and not VCO2, which is more closely linked with breathing? Please add a graph of VO2 in the different conditions. That is very important variable in a small mammal, and its regulation could be altered by beta amyloid in the wrong place.

Weve added both VCO2 and VO2 for all conditions as a supplemental figure. While we have graphed the VO2 for each condition, we want to emphasize the hypoxia metabolic response may be misleading. We examined the first part of the HVR (ventilatory rise, first 5 minutes of the response) and not the metabolic decline that follows. (see next comment). That said, male mice did have noticeable differences in resting VCO2 and VO2 if the resting values were presented separately by exposure type. If all resting values were grouped (table 1) then that difference did not exist. We've updated the discussion accordingly to briefly state that little metabolic effects were seen with our analysis.

• Were there any differences in temperature or metabolic responses to hypoxia between the strains?

We tested the first phase of the HVR which is why we only use a 5-minute exposure to capture the ventilatory response before the second phase, metabolic response comes in. The differences we did see in hypoxia were due namely to higher resting values in 5XFAD male mice only in the hypoxia exposure trials. We tried to reflect that in the discussion. We do not report any differences in body temperature responses given the short nature of the exposure. Metabolic information was added (see previous comment) but not temperature.

While the HMR (metabolic response to hypoxia is interesting) we wanted to focus on the ventilatory aspect of the response only for this study. Primarily given the difference in the HMR between humans and mice.

• The chemoreflex blunting is important, yet not as profound as reported in a Tau model of AD. Is this potentially due to differences in tau vs amyloid, or age or other factors?

We added some discussion on this. The degree of tauopathy and amyloidosis is not controlled for when comparing these studies. Thus, age and pathological progression likely account for these discrepancies. A temporal study

in these models would be hugely beneficial, particularly the 5XFAD model given the age dependent beta amyloidosis.

Re: JP-RP-2026-290936R1 "**Alzheimer's disease: Amyloid beta plaque pathology induces chemosensory deficits and increases apneas in the 5XFAD mouse model of Alzheimer's disease** " by Ryan J Sprenger, Andrea C Ewald, Phinea Z Romero, Kaitlyn M Marino, Maia G Gumnit, Erin C McCann, Abigail B Radcliff, Tyler K Ulland, Jyoti Watters, and Tracy L Baker

Dear Dr Sprenger,

Thank you for submitting your manuscript to The Journal of Physiology. It has been assessed by a Reviewing Editor and by 2 expert referees and we are pleased to tell you that it is potentially acceptable for publication following satisfactory major revision.

Please advise your co-authors of this decision as soon as possible.

The referee reports are copied at the end of this email.

Please address all the points raised and incorporate all requested revisions or explain in your Response to Referees why a change has not been made. We hope you will find the comments helpful and that you will be able to return your revised manuscript within 2 months. If your article is NOT for a Special Issue, you may have 9 months to revise. If you require an extension, please contact journal staff: jp@physoc.org. Please note that this letter does not constitute a guarantee for acceptance of your revised manuscript.

Your revised manuscript should be submitted online using the link in your Author Tasks: Link Not Available. This link is accessible via your account as Corresponding Author; it is not available to your co-authors. If this presents a problem, please contact journal staff (jp@physoc.org). Image files from the previous version are retained on the system. Please ensure you replace or remove any files that are being revised.

If you do not wish to submit a revised version of your manuscript, you must inform our journal staff (jp@physoc.org) or reply to this email to request withdrawal. Please note that a manuscript must be formally withdrawn from the peer review process at one journal before it may be submitted to another journal.

TRANSPARENT PEER REVIEW POLICY: To improve the transparency of its peer review process, The Journal of Physiology publishes online as supporting information the peer review history of all articles accepted for publication. Readers will have access to decision letters, including Editors' comments and referee reports, for each version of the manuscript, as well as any author responses to peer review comments. Referees can decide whether or not they wish to be named on the peer review history document.

ABSTRACT FIGURES: Authors are expected to use The Journal's premium BioRender account to create/redraw their Abstract Figures. Information on how to access this account is here: https://physoc.onlinelibrary.wiley.com/journal/14697793/biorender-access.

This will enable Authors to create and download high-resolution figures. If authors have used the free BioRender service, they can use the instructions provided in the link above to download a high-resolution version suitable for publication.

The link provided should only be used for the purposes of this submission. Authors will be charged for figures created on this account if they are not related to this manuscript submission.

LANGUAGE EDITING AND SUPPORT FOR PUBLICATION: If you would like help with English language editing, or other article preparation support, Wiley Editing Services offers expert help, including English Language Editing, as well as translation, manuscript formatting, and figure formatting at www.wileyauthors.com/eeo/preparation. You can also find resources for Preparing Your Article for general guidance about writing and preparing your manuscript at www.wileyauthors.com/eeo/prepresources.

RIGOUR AND REPRODUCIBILITY: We recommend authors consult The Journal's Rigour and Reproducibility requirements to ensure that their manuscript is suitable for publication: https://physoc.onlinelibrary.wiley.com/pb-assets/hub-assets/physoc/documents/TJP-Rigour-and-Reproducibility-Requirements.pdf

REVISION CHECKLIST:

Check that your Methods section conforms to journal policy: https://jp.msubmit.net/cgi-bin/main.plex?form_type=display_requirements#methods.

Check that data presented conforms to the statistics policy: https://jp.msubmit.net/cgi-bin/main.plex?form_type=display_requirements#statistics.

Upload a full Response to Referees file. To create your 'Response to Referees': copy all the reports, including any comments from the Senior and Reviewing Editors, into a Microsoft Word, or similar, file and respond to each point, using

font or background colour to distinguish comments and responses and upload as the required file type.

Please upload two versions of your manuscript text: one with all relevant changes highlighted and one clean version with no changes tracked. The manuscript file should include all tables and figure legends, but each figure/graph should be uploaded as separate, high-resolution files.

You may also upload:

- 'Potential Cover Art' for consideration as the issue's cover image
- Appropriate Supporting Information (Video, audio or data set: see https://jp.msubmit.net/cgi-bin/main.plex?form_type=display_requirements#supp).

We look forward to receiving your revised submission.

If you have any queries, please reply to this email and we will be pleased to advise.

Yours sincerely,

Bettina Mittendorfer
Senior Editor
The Journal of Physiology

REQUIRED ITEMS

- I am aware that you have faced technical issues with BioRender. Once these are resolved, please submit your abstract figure.

EDITOR COMMENTS

Reviewing Editor:

Thank you for the extensive revisions made to the manuscript. However, Reviewer 2 has raised several thoughtful and constructive points regarding the accuracy of the discussion of your findings and the appropriateness of certain citations. These issues must be addressed prior to final acceptance. Please carefully consider all of Reviewer 2's comments and revise the manuscript accordingly. I look forward to receiving your revised manuscript.

REFEREE COMMENTS

Referee #1:

The authors did a great job addressing all reviewer concerns and i have no additional concerns.

Referee #2:

Overall, the authors improved the manuscript, but there remain many points where the writing may best be described as sloppy or even "flippant" with respect to conclusions possible from experimental findings or in misquoting literature. The straight-forward question framing this study is: does progressive amyloidosis impact breathing stability and chemoreflexes in mice. That should be discussed to the fullest without equating it with AD at each stage. AD can be used to introduce and to exit the paper but mixing the two throughout makes the manuscript misleading.

My biggest objections concern (writing) confusion that seems to:

• Equate human AD with different murine models of beta amyloid, tau or other brain pathologies. It is not in fact clear that any of these murine models are truly AD, and the paper should be written with discussion of human AD distinct from the findings of beta amyloid over-expression studied here.

• Confuse the link between human obstructive sleep apnea and the impact of either exogenously applied chronic intermittent hypoxia in murine models or the endogenous hypoxic hypercapnia (undocumented) in the beta amyloid mice. Mice do not have obstructive sleep apnea since they have bony support of the upper airways that is not present in humans. Thus, the apneas reported here are central in nature, and produce intermittent hypoxia or hypercapnia of an unspecified level.

• Many papers are not cited correctly-they are often cited for a feature that is not inherent in the paper cited. For example, it is stated in the intro: Intermittent hypoxia exacerbates AD symptom progression in the 3xTg AD mouse model of Aß plaque deposition (Shiota et al., 2013). Although the Shihota study uses exogenous CIH in transgenic mice, it cannot be equated with the impact of the genetic manipulation alone, as in this study-at least not without further qualification. We don't know if their apneas reported here are enough to do anything alone. It can be speculated if clearly labeled. I suggest looking at all references to be sure they are correctly used.

• Some of the responses to the reviewer from the prior round are a bit "flip" suggesting that command of the English language is at fault for saying that loop gain decreased rather than increased or vice versa at several points in the manuscript. This raises concern. On a small scale, the following passage suffers from that confusion more subtly: central sleep apnea (CSA) arises from impairments in respiratory drive most often due to hypersensitivity in chemosensory feedback and central respiratory function. The term dysfunction may be more accurate since the word impairment implies a loss of function. Increased gain is the most common source of impairment, not decrement. Although it is stated that others suggest low loop gain can cause instability, there are scarcely any convincing papers cited.

• This statement is not correct: The present study describes the occurrence of apneas in the 5XFAD mouse model, a control of breathing pathology not previously described in other AD models. It has been described in other mouse models as was pointed out in review.

• I find this statement reasonably compelling: In the present study, reduced chemosensory responses suggest a low-loop gain situation where chemosensors fail to adequately respond to changes in blood gases, rather than high-loop gain (Dempsey, 2019). That statement is interesting but could be elaborated keeping the above concerns in mind.

END OF COMMENTS

Response to reviewer: Revision 2

The authors would like to thank reviewer 2 for the additional comments. We have addressed all comments below, and incorporated edits into the manuscript for those which we believe would strengthen or clarify. Some comments, particularly those concerning contextualization of why we examined or discussed certain parameters related to Alzheimer's disease, we felt, would be deleterious to the manuscript, so those were not incorporated. Please see specific responses to all comments below, in blue.

Overall, the authors improved the manuscript, but there remain many points where the writing may best be described as sloppy or even "flippant" with respect to conclusions possible from experimental findings or in misquoting literature. The straight-forward question framing this study is: does progressive amyloidosis impact breathing stability and chemoreflexes in mice. That should be discussed to the fullest without equating it with AD at each stage. AD can be used to introduce and to exit the paper but mixing the two throughout makes the manuscript misleading.

We thank the reviewer for recognizing the improvement to our manuscript.

Before addressing the scientific aspects of this critique, we wish to express our disagreement with the characterization that our writing is "flippant or sloppy". This comment, in the absence of specific examples, seems unfounded and does not maintain the level of professionalism expected in the manuscript review process. Notwithstanding, please see specific responses below.

My biggest objections concern (writing) confusion that seems to:

• Equate human AD with different murine models of beta amyloid, tau or other brain pathologies. It is not in fact clear that any of these murine models are truly AD, and the paper should be written with discussion of human AD distinct from the findings of beta amyloid over-expression studied here.

We have re-examined the manuscript to ensure that there are no phrases doing so. It is quite clear that animal models of AD are just that, models, that do not directly mimic the disease, which is of course true for nearly every animal model of any human disease. However, amyloidosis is indeed a hallmark of AD, as is tauopathy, and to not mention these aspects would be misleading, especially when the reason we chose the 5XFAD model of amyloidosis was because of its role in human AD. We have checked the manuscript again and found no wording stating that murine models are AD. Where appropriate, we utilized phrasing such as "AD-like", amyloidosis, or tauopathy. Despite this, we softened our phrasing even further in a few places.

We respectfully disagree that removal of AD discussion aside from the introduction and discussion would benefit this manuscript. The way we discuss the 5XFAD model at the very least meets the current accepted standard for this murine model of AD and other models. Where we do discuss human AD allows us to contextualize why we examined or discussed a certain parameter. For example, in our “Sleep disordered breathing and Alzheimer’s disease” section in the discussion, we utilize information from human AD studies to bring context as to why we would examine for SDB in this model, what would be the result of SDB (hypoxia), and how hypoxia is known to alter AD progression in humans.

Finally, in reference to the point above, we would like to highlight two recent papers published in the Journal of Physiology in which the 5XFAD model is discussed less conservatively in the context of AD (one of which is even titled with Alzheimer’s disease, with no mention of the animal model used). We agree with the reviewer that the 5XFAD model and other rodent models are not AD directly, but it is important to make the point that we do not discuss this model any differently than how it is discussed in the extensive 5XFAD literature. The comments by the reviewer would have us meet a widely different standard than currently accepted in the field, and in this journal itself.

- https://pmc.ncbi.nlm.nih.gov/articles/PMC12148202/#tjp16755-sec-0150
- https://physoc.onlinelibrary.wiley.com/doi/10.1113/JP289018

• Confuse the link between human obstructive sleep apnea and the impact of either exogenously applied chronic intermittent hypoxia in murine models or the endogenous hypoxic hypercapnia (undocumented) in the beta amyloid mice. Mice do not have obstructive sleep apnea since they have bony support of the upper airways that is not present in humans. Thus, the apneas reported here are central in nature, and produce intermittent hypoxia or hypercapnia of an unspecified level.

We re-examined the manuscript to ensure that we do not state that the model we examined had OSA. In fact, in lines 299-302 we specifically stated that the apneas we observed were CSAs. We do, in those lines, qualify that under very specific circumstances (namely obesity models) that some form of OSA can occur in mice, but that this was likely not the case in this study.

We have tried to adjust the manuscript to make it clear that: 1) the result of OSA is hypoxia in humans which aggravates AD progression, 2) CIH (designed to model the hypoxia from OSA) aggravates pathology in murine models, and 3) the apneas in our model (while we don’t measure blood oxygen, which we state) likely results in hypoxia that could aggravate amyloidosis. This is all to say that results from this model suggest that amyloidosis in humans may be exacerbated by SDB and enhance disease progression.

We also edited the manuscript to clarify the link between CSA and OSA in humans, and that the CSAs in this model might inform on the development of OSA in humans (example (lines 80-82, and 303-316).

• Many papers are not cited correctly-they are often cited for a feature that is not inherent in the paper cited. For example, it is stated in the intro: Intermittent hypoxia exacerbates AD symptom progression in the 3xTg AD mouse model of Aß plaque deposition (Shiota et al., 2013). Although the Shihota study uses exogenous CIH in transgenic mice, it cannot be equated with the impact of the genetic manipulation alone, as in this study-at least not without further qualification. We don't know if their apneas reported here are enough to do anything alone. It can be speculated if clearly labeled. I suggest looking at all references to be sure they are correctly used.

We have gone through the manuscript to confirm papers are cited correctly.

However, we are a bit confused by the specific example in this comment. Is the reviewer suggesting that, because Shiota et al used a genetic model of AD, that the results of IH exacerbated amyloidosis cannot be interpreted as such? The authors in that manuscript provide evidence that when given chronic intermittent hypoxia, 3xTg-AD mice display an increase in AB42 compared to mice not exposed to CIH. We cite this paper and summarize their conclusion, which is that the intermittent hypoxia caused an increase in amyloidosis in 3xTg-AD mice, suggesting that hypoxia aggravates amyloid progression. This accurately represents the data and the authors' conclusion.

• Some of the responses to the reviewer from the prior round are a bit "flip" suggesting that command of the English language is at fault for saying that loop gain decreased rather than increased or vice versa at several points in the manuscript. This raises concern. On a small scale, the following passage suffers from that confusion more subtly: central sleep apnea (CSA) arises from impairments in respiratory drive most often due to hypersensitivity in chemosensory feedback and central respiratory function. The term dysfunction may be more accurate since the word impairment implies a loss of function. Increased gain is the most common source of impairment, not decrement. Although it is stated that others suggest low loop gain can cause instability, there are scarcely any convincing papers cited.

We are unsure of the implication of "flip" in this comment. If the reviewer is referencing the initial "flippant" description of our writing, then we want to stress that we did not intend to be dismissive of the reviewers' original comments. And, that we corrected our mis-statements on low vs high loop gain from the original manuscript. It is fair to say, as the reviewer points out

here, that some of the misconceptions may have been due to word usage that could be perceived in multiple ways. For example, impairment versus dysfunction. Impairment does not necessarily mean reduced by nature (definition: loss of or abnormality in organ function). We used it with the latter context, the reviewer suggests the former.

If the reviewer is suggesting that we have "flipped" how we now discuss low and high loop gain, then we would like to point out that this was in response to the request of this reviewer, if this is the intent of this comment.

Finally, regarding low loop gain evidence. We entirely agree that the majority of apneas are the result of high loop gain. And we specifically stated this in lines 336-337, 356-357 and 472. We do, however, also examine the literature in our Discussion section for instances of low loop gain being related to CSA, to help contextualize why we may have observed our results. It is important to discuss multiple explanations/possibilities for a given result, even if they are different from (or even contrary to) our own preconceived notions. We also discuss a tau mouse model of AD which had similar reductions in chemosensitivity. However, we wish to stress that in the manuscript, we make it clear that while high loop gain is the typical culprit of reduced chemosensitivity, that in some cases, low loop gain can also be the cause.

• This statement is not correct: The present study describes the occurrence of apneas in the 5XFAD mouse model, a control of breathing pathology not previously described in other AD models. It has been described in other mouse models as was pointed out in review.

We have corrected this error, thank you for bringing it to our attention. The reviewer is correct, there are models where this pathology has been shown, and we now reference them appropriately. We have also ensured this was not mis-stated elsewhere in the manuscript.

• I find this statement reasonably compelling: In the present study, reduced chemosensory responses suggest a low-loop gain situation where chemosensors fail to adequately respond to changes in blood gases, rather than high-loop gain (Dempsey, 2019). That statement is interesting but could be elaborated keeping the above concerns in mind.

We have added wording on this (lines 343-351) in addition to other discussion of this topic in lines (352-360). We find this observation interesting as well, and worthy of further investigation.

Dear Dr Sprenger,

Re: JP-RP-2026-290936R2 "**Alzheimer's disease: Amyloid beta plaque pathology induces chemosensory deficits and increases apneas in the 5XFAD mouse model of Alzheimer's disease** " by Ryan J Sprenger, Andrea C Ewald, Phinea Z Romero, Kaitlyn M Marino, Maia G Gumnit, Erin C McCann, Abigail B Radcliff, Tyler K Ulland, Jyoti Watters, and Tracy L Baker

Thank you for submitting your manuscript to The Journal of Physiology. It has been assessed by a Reviewing Editor and by 2 expert referees and we are pleased to tell you that it is acceptable for publication following satisfactory revision.

The referee reports are copied at the end of this email.

Please address all the points raised and incorporate all requested revisions or explain in your Response to Referees why a change has not been made. We hope you will find the comments helpful and that you will be able to return your revised manuscript within 4 weeks. If you require longer than this, please contact journal staff: jp@physoc.org.

Your revised manuscript should be submitted online using the link in your Author Tasks Link Not Available. This link is accessible via your account as Corresponding Author; it is not available to your co-authors. If this presents a problem, please contact journal staff (jp@physoc.org). Image files from the previous version are retained on the system. Please ensure you replace or remove any files that are being revised.

If you do not wish to submit a revised version of your manuscript, you must inform our journal staff (jp@physoc.org) or reply to this email to request withdrawal. Please note that a manuscript must be formally withdrawn from the peer review process at one journal before it may be submitted to another journal.

TRANSPARENT PEER REVIEW POLICY: To improve the transparency of its peer review process The Journal of Physiology publishes online, as supporting information, the peer review history of all articles accepted for publication. Readers will have access to decision letters, including Editors' comments and referee reports, for each version of the manuscript, as well as any author responses to peer review comments. Referees can decide whether or not they wish to be named on the peer review history document.

ABSTRACT FIGURES: Authors are expected to use The Journal's premium BioRender account to create/redraw their Abstract Figures. Information on how to access this account is here: https://physoc.onlinelibrary.wiley.com/journal/14697793/biorender-access.

This will enable Authors to create and download high-resolution figures. If authors have used the free BioRender service, they can use the instructions provided in the link above to download a high-resolution version suitable for publication.

The link provided should only be used for the purposes of this submission. Authors will be charged for figures created on this account if they are not related to this manuscript submission.

LANGUAGE EDITING AND SUPPORT FOR PUBLICATION: If you would like help with English language editing, or other article preparation support, Wiley Editing Services offers expert help, including English Language Editing, as well as translation, manuscript formatting, and figure formatting at www.wileyauthors.com/eeo/preparation. You can also find resources for Preparing Your Article for general guidance about writing and preparing your manuscript at www.wileyauthors.com/eeo/prepresources.

RIGOUR AND REPRODUCIBILITY: We recommend authors consult The Journal's Rigour and Reproducibility requirements to ensure that their manuscript is suitable for publication: https://physoc.onlinelibrary.wiley.com/pb-assets/hub-assets/physoc/documents/TJP-Rigour-and-Reproducibility-Requirements.pdf

REVISION CHECKLIST:

Check that your Methods section conforms to journal policy: https://jp.msubmit.net/cgi-bin/main.plex?form_type=display_requirements#methods

Check that data presented conforms to the statistics policy: https://jp.msubmit.net/cgi-bin/main.plex?form_type=display_requirements#statistics

Upload a full Response to Referees file. To create your 'Response to Referees' copy all the reports, including any comments from the Senior and Reviewing Editors, into a Microsoft Word, or similar, file and respond to each point, using font or background colour to distinguish comments and responses and upload as the required file type.

Please upload two versions of your manuscript text: one with all relevant changes highlighted and one clean version with no changes tracked. The manuscript file should include all tables and figure legends, but each figure/graph should be uploaded as separate, high-resolution files. The journal is now integrated with Wiley's Image Checking service. For further details,

see: https://www.wiley.com/en-us/network/publishing/research-publishing/trending-stories/upholding-image-integrity-wileys-image-screening-service

You may also upload:

- 'Potential Cover Art' for consideration as the issue's cover image
- Appropriate Supporting Information (Video, audio or data set: see https://jp.msubmit.net/cgi-bin/main.plex?form_type=display_requirements#supp).

We look forward to receiving your revised submission.

If you have any queries, please reply to this email and we will be pleased to advise.

Yours sincerely,

Bettina Mittendorfer
Senior Editor
The Journal of Physiology

EDITOR COMMENTS

Reviewing Editor:

Thank you for your extensive revision. The reviewers have come down in favor of publication, noting that the study is solid and worth publishing. However, minor revisions are required regarding citation accuracy. Please revise the introduction to ensure that your claims about chemosensory dysfunction and sleep-disordered breathing are properly supported by the cited references. I look forward to your final version.

REFEREE COMMENTS

Referee #1:

I commend the authors on an interesting study that is worthy of publication in J Physiol

Referee #2:

Overall, this paper has been improved.

END OF COMMENTS

Response to reviewer: Revision 3

We would like to thank all the reviewers and the reviewing editor for helping make this manuscript better. Please see specific comments to this round of minor edits below in blue.

Reviewing Editor:

Thank you for your extensive revision. The reviewers have come down in favor of publication, noting that the study is solid and worth publishing. However, minor revisions are required regarding citation accuracy. Please revise the introduction to ensure that your claims about chemosensory dysfunction and sleep-disordered breathing are properly supported by the cited references. I look forward to your final version.

Thank you for pointing out where the citations could be better utilized to support the claims we make in the introduction. We have edited one area of citations heavily regarding chemosensory dysfunction and SBD in the introduction:

- Lines 90-91 and several locations in the citation list: We have added several citations to this in addition to the Mulkey paper we originally cited after stating there is growing appreciation of chemosensory dysfunction in disease. Below is a list of citations with the reason why it was added:
    - Babiloni et al. 2014 adds evidence of hypercapnia influence on cerebral vasomotor reactivity and EEG coherence in MCI patients suggesting changes in CO_2 status affects function
    - Burtscher et al. 2024 adds evidence of hypoxia sensing/dysfunction in Parkinson's Disease adding another neurogenerative disease to this claim
    - Cabot et al. 2026 adds evidence of astrocyte hypoxia sensing and its role in AD, adding another pathway of oxygen sensing dysfunction and its role in AD progression
    - Correia and Moreira 2021 adds enhanced evidence of hypoxia sensing and signaling dysfunction in AD
    - Erhardt et al. 2025 glymphatic clearing relies in part on CO_2 status highlighting the role of maintained co2 sensing in disease progression in Parkinson's Disease
    - Pokusa et al. 2019 is a review centered respiratory dysfunction in Parkinson's Disease, and it examines in parts the influence of chemoreception dysfunction in this pathogenesis
    - Rey et al. 2023 is a review on oxygen sensing in neurodegenerative disease

For the in-text citations after the above list (lines 93-94) we examined which citations we used and why, and decided not to change this list for the following reasons for each:

- Iseki et al 1989, this was one of the first reports of AD pathology (AB plaques) in the brainstem which is a major cite of not only chemoreception but also respiratory control resulting from this sensing.
- Simic et al. 2009 poses the question of where AD pathology first shows up and reviews evidence of AB in the brainstem. For example, the pons and medulla (sites of chemoreception) and specifically the locus coeruleus a major site of chemoreception are examined. This provides evidence of AB deposition in major sites of chemoreception and control.
- Gaig and Ironzo 2012 examine the incidence and nature of SDB in neurodegenerative diseases
- Orr 2017 examines in part the role of chemosensory dysfunction in CSA and complex sleep apnea specifically.
- Dempsey 2019 examines chemosensory dysfunction in CSA
- Humphrey et al. 2022 gives an example of a model of AD (STZ) and how it causes progression of disease in the NTS a major site of chemosensing and respiratory control showing that in this model, AD-like pathology results in chemosensory dysfunction.

With all of the introduction citations in mind, we feel that it now provides a nice progression of evidence that 1) hypoxia and hypoxia from OSA, CSA, complex sleep apnea result in at least disease aggravation in AD but also potentially onset of disease (lines 72-77), 2) many AD patients have some sort of SDB (lines 75-77), 3) chemosensory dysfunction is often part of the pathogenesis for SDB (lines 81, 83, and 94), 4) chemosensory dysfunction is also present and potentially contributing in/to neurodegenerative disease (lines 90-91), and finally 5) AD progression in major sites of central chemosensory control as well as evidence of synaptic loss due to AD-like progression in the NTS and LC which are important to chemosensing and respiratory control. Finally, we cite evidence of AB progression in human brainstem tissue and the timing of that progression compared to the cortical tissue giving more evidence that SDB may be involved in the onset of disease.

The bibliography has edited now to reflect the addition of the new citations.

REFEREE COMMENTS

Referee #1:

I commend the authors on an interesting study that is worthy of publication in J Physiol
Thank you for your kind words and help with bettering this manuscript.

Referee #2:

Overall, this paper has been improved.

Thank you for helping improve this manuscript.

3rd Revision - Editorial Decision **06-Aug-2026**

Dear Dr Sprenger,

Re: JP-RP-2026-290936R3 "**Alzheimer's disease: Amyloid beta plaque pathology induces chemosensory deficits and increases apneas in the 5XFAD mouse model of Alzheimer's disease** " by Ryan J Sprenger, Andrea C Ewald, Phinea Z Romero, Kaitlyn M Marino, Maia G Gumnit, Erin C McCann, Abigail B Radcliff, Tyler K Ulland, Jyoti Watters, and Tracy L Baker

We are pleased to tell you that your paper has been accepted for publication in The Journal of Physiology.

The last Word (or similar) version of the manuscript provided will be used by the Production Editor to prepare your proof. When this is ready you will receive an email containing a link to Wiley's Online Proofing System. The proof should be thoroughly checked and corrected as promptly as possible.

Authors should note that it is too late at this point to offer corrections prior to proofreading. Major corrections at proof stage, such as changes to figures, will be referred to the Editors for approval before they can be incorporated. Only minor changes, such as to style and consistency, should be made at proof stage. Changes that need to be made after proof stage will usually require a formal correction notice.

All queries at proof stage should be sent to: TJP@wiley.com.

Following copyediting and typesetting the accepted version will be published online.

Yours sincerely,

Bettina Mittendorfer
Senior Editor
The Journal of Physiology

IMPORTANT POINTS TO NOTE FOLLOWING ACCEPTANCE OF YOUR PAPER:

• IMPORTANT NOTICE ABOUT OPEN ACCESS: To assist authors whose funding agencies mandate immediate public access to published research findings, The Journal of Physiology allows authors to pay an Open Access (OA) fee to have their papers made freely available immediately on publication.

The Corresponding Author will receive an email from Wiley with details on how to register or log in to Wiley Authors where you will be able to place an order.

• You can check if your funder or institution has a Wiley Open Access Account here:
https://authors.wiley.com/author-resources/Journal-Authors/open-access/author-compliance-tool.html

MEMBER DISCOUNT: Corresponding authors who are Members of the Physiological Society are eligible for a 20% discount on the cost of the Article Processing Charge (APC). To claim this discount, the Corresponding Author will need to log in to the Physiological Society Membership portal and retrieve the discount code (https://www.physoc.org/journals-and-media/journals/journal-discount-for-members/). This code should then be quoted in the billing section of Wiley Authors when selecting the open access option.

• TRANSPARENT PEER REVIEW POLICY: To improve the transparency of its peer review process, The Journal of Physiology publishes online as supporting information the peer review history of all articles accepted for publication. Readers will have access to decision letters, including Editors' comments and referee reports, for each version of the manuscript as well as any author responses to peer review comments. Referees can decide whether or not they wish to be named on the peer review history document.

• You can help your research get the attention it deserves! Check out Wiley's free Promotion Guide for best-practice recommendations for promoting your work at: www.wileyauthors.com/eeo/guide. You can learn more about Wiley Editing Services which offers professional video, design, and writing services to create shareable video abstracts, infographics, conference posters, lay summaries, and research news stories for your research at: www.wileyauthors.com/eeo/promotion.

• If you would like to receive our 'Research Roundup', a monthly newsletter highlighting the cutting-edge research published in The Physiological Society's family of journals (The Journal of Physiology, Experimental Physiology, Physiological Reports, The Journal of Nutritional Physiology and The Journal of Precision Medicine: Health and Disease), please click this link, fill in your name and email address and select 'Research Roundup':

https://www.physoc.org/journals-and-media/membernews

EDITOR COMMENTS

Reviewing Editor:

Thank you for making the revision. I have no further questions.
